# Impact of Soil Fertilization with Pig Slurry on Antibiotic Residues and Resistance Genes: A Longitudinal Study

**DOI:** 10.3390/antibiotics13060486

**Published:** 2024-05-24

**Authors:** Luisa Massaccesi, Elisa Albini, Francesca Romana Massacci, Danilo Giusepponi, Fabiola Paoletti, Stefano Sdogati, Francesco Morena, Alberto Agnelli, Angelo Leccese, Chiara Francesca Magistrali, Roberta Galarini

**Affiliations:** 1 National Research Council of Italy, Institute for Agriculture and Forestry Systems in the Mediterranean (ISAFOM-CNR), 06128 Perugia, Italy; luisa.massaccesi@cnr.it; 2 Istituto Zooprofilattico Sperimentale dell’Umbria e delle Marche “Togo Rosati”, 06126 Perugia, Italy; e.albini@izsum.it (E.A.); d.giusepponi@izsum.it (D.G.); f.paoletti@izsum.it (F.P.); stefano.sdogati@izsum.it (S.S.); c.magistrali@izsler.it (C.F.M.); r.galarini@izsum.it (R.G.); 3 Department of Chemistry, Biology and Biotechnology, Biochemical and Biotechnological Sciences, University of Perugia, 06122 Perugia, Italy; francesco.morena@unipg.it; 4 Department of Agricultural, Food and Environmental Science, University of Perugia, 06124 Perugia, Italy; alberto.agnelli@unipg.it (A.A.); angelo.leccese@unipg.it (A.L.); 5 Istituto Zooprofilattico Sperimentale della Lombardia e dell’Emilia Romagna “Bruno Ubertini”, 25124 Brescia, Italy

**Keywords:** manure treatment, antibiotic residues, antibiotic resistance genes, swine, slurry

## Abstract

The impact of soil fertilization with animal manure on the spread and persistence of antibiotic resistance in the environment is far from being fully understood. To add knowledge about persistence and correlations between antibiotic residues and antibiotic resistance genes (ARGs) in fertilized soil, a longitudinal soil mesocosm study was conducted. Soil samples were collected from the mesocosms immediately before spreading and then afterward at fifteen time points during a 320-day observation period. Eight ARGs (*ermB*, *sul1*, *tet*A, *tet*G, *tet*M, *cfr*, *fex*A, and *optr*A) and the class 1 integron-integrase gene, *intI1*, were determined in both pig slurry and soil, as well as residues of 36 antibiotics. Soil chemical and biochemical parameters were also measured. Twelve antibiotics were detected in the slurry in the range of 3 µg kg^−1^–3605 µg kg^−1^, with doxycycline, lincomycin, and tiamulin being the most abundant, whereas *ermB*, *sul1*, and *tetM* were the predominant ARGs. Before spreading, neither antibiotic residues nor ARGs were detectable in the soil; afterwards, their concentrations mirrored those in the slurry, with a gradual decline over the duration of the experiment. After about three months, the effect of the amendment was almost over, and no further evolution was observed.

## 1. Introduction

Antibiotic resistance is a biologically adaptive phenomenon of microorganisms that manage to survive or grow in the presence of an antibiotic substance. The presence of antibiotic resistance genes (ARGs) in the environment contributes to the dissemination of “multi-drug resistant” pathogens insensitive to common pharmacological treatments, posing concerns for animal and human health. Antibiotic residues and resistance genes can enter the environment through wastewater treatment plants (WWTPs) and healthcare facility sewage, but a considerable amount is ascribable to farming practices. The use of manure for soil amendment is a very common agricultural practice that, other than having a positive effect on soil fertility, represents a critical aspect in the environmental diffusion of ARGs, resistant bacterial strains, antibiotics, and their transformation products [1,2]. Around 30–90% of the antibiotics administered to livestock breeding are excreted as unchanged drugs or active metabolites from animal organisms [3]. Therefore, soils amended with zootechnical residues (manure, slurry, poultry litter, etc.) are recognized as reservoirs of ARGs potentially transmissible to soil-harboring bacteria and pathogens [4] and to the vegetable microbiome [5]. The vast market demand for antibiotics and their inappropriate use globally have increased the spread of ARGs in the soil microbial community.

Although several papers have been published in recent years adding information on the presence and correlations between antibiotic residues, ARGs, and physicochemical parameters of amended soils, several aspects still remain contradictory and worthy of further investigation [1,6]. The lack of agreement between some scientific data depends on the high number of variables affecting this phenomenon, such as the type of antibiotics administered, animal species, frequency of antibiotic treatments, animal metabolism, manure storage conditions and their distribution to agricultural fields, soil properties, etc. [6,7]. It has also been proven that the presence of various environmental pollutants, such as heavy metals, pesticides, and polycyclic aromatic hydrocarbons, can increase ARG concentrations [8]. In addition, although chemical and biomolecular methodologies have been refined, the implementation of procedures able to detect, at the same time, several antibiotic classes and tens of ARGs in very complex matrices such as manure and soil is still challenging, and quantitative data can be affected by large uncertainties [9].

Seven classes of antibiotics (lincosamides, macrolides, phenicols, pleuromutilins, quinolones, sulphonamides, and tetracyclines) were quantified by liquid chromatography coupled to high-resolution mass spectrometry (LC-HRMS) in slurry and soil throughout the entire experiment duration. In the same samples, a class 1 integron-integrase gene, *IntI1*, and eight ARGs (*ermB*, *sul1*, *tetA*, *tetG*, *tetM*, *cfr*, *fexA*, and *optrA*) were determined by quantitative PCR technique as well as soil chemical and biochemical parameters (pH, total organic carbon, total nitrogen, soluble organic form of carbon and nitrogen, microbial biomass carbon and nitrogen, basal soil respiration). 

The hypothesis behind this study is that ARGs and antibiotic residues persist in soil after amending with pig slurry and contribute to the spread of resistant bacteria. To confirm this hypothesis, and since the pig industry is considered one of the main production systems using antibiotics in Europe, we decided to perform a longitudinal soil mesocosm study using pig slurry belonging to a conventional farm. 

## 2. Results

### 2.1. Soil Analysis

The mean values of chemical and biochemical parameters measured in amended and control soil are shown in Table 1. The treated soil was analysed at each of the fifteen time points, whereas the control soil (unamended soil) was analysed only at t1, t6, t12, and t15. The addition of pig slurry (pH 7.20) led to a significant decrease in soil pH from 7.90 (t1 of control soil) to 6.87 (t1 of treated soil). After about one month (t6), the pH value rose to 7.32, and then it remained quite stable in the range of 7.31–7.58 for the whole experiment. However, throughout the duration of the experiment, pH values were always significantly higher in the treated soil than in the control one. Immediately after the amendment, total organic carbon (TOC), total nitrogen (TN), and the total soluble form of nitrogen (TSN) increased and remained almost constant until the last time point (Table 1). Differently, no significant increase was observed for water-soluble organic carbon (WSOC) before and after the spreading. During the first three months (t1–t8), the contents of microbial carbon (C_mic_) and nitrogen (N_mic_) in the treated soil were markedly higher than those measured in the untreated samples. Later (t9–t15), their concentrations decreased and became progressively comparable to those measured in the control soil. The pig slurry treatment did not noticeably affect the mineralogical assemblage of the whole soil, although an increase in calcite and a decrease in plagioclases occurred in the sand fraction (Table S7). 

### 2.2. Antibiotics in Pig Manure and Soil

The choice of antibiotics to be included in the analytical method was made considering the drugs most commonly administered in Umbrian swine farming. Veterinary prescriptions recorded at the pig farm where the slurry was collected were also consulted. All the antibiotics used were, therefore, included in the developed LC-HRMS method (Figure S1), with two important exceptions: β-lactams (penicillins and cephalosporins), which are known to degrade rapidly in contact with manure [3], and aminoglycosides, the determination of which in complex matrices is very problematic [10]. Chromatograms of all the determined antibiotics are reported in the Supplementary Material (Figures S2–S7). 

**Figure 1:**
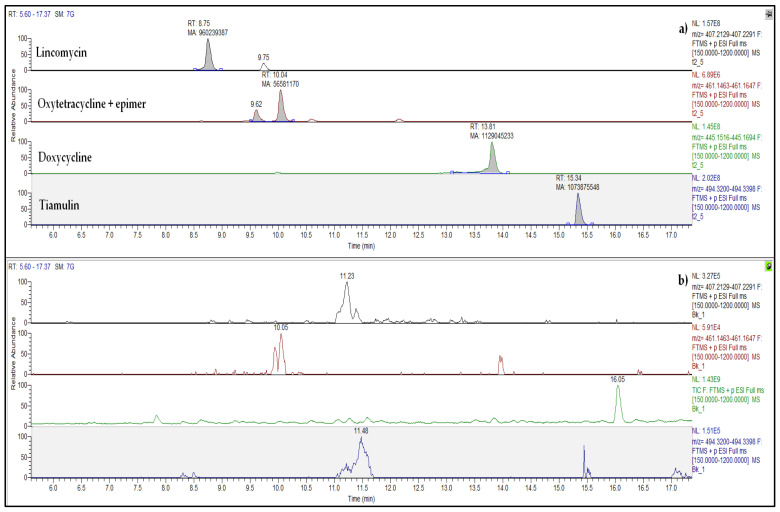
Extracted ion chromatograms: (**a**) soil after two days of spreading (t2); (**b**) soil before slurry spreading (t0). The peaks of the four most abundant antibiotics (lincomycin, oxytetracycline, doxycycline, and tiamulin) are shown.

**Figure 2:**
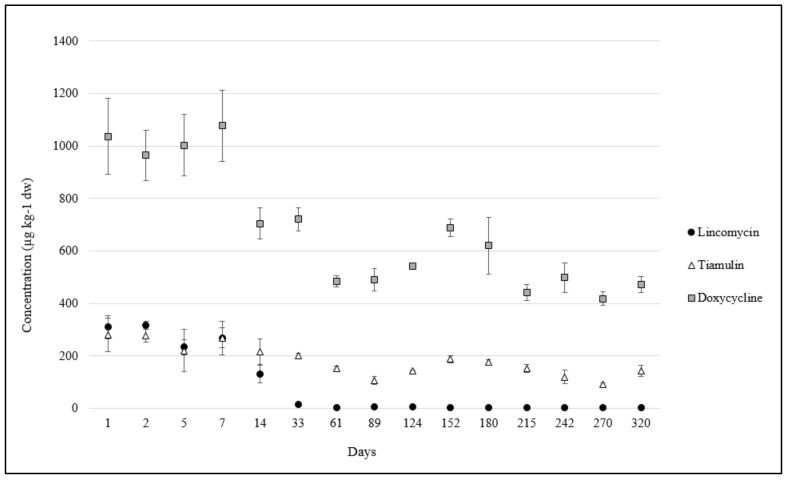
Soil concentrations (µg kg^−1^ dw) of doxycycline (DOX), tiamulin (TIA), and lincomycin (LIN) during the incubation experiment.

In the pig slurry collected at the local farm and used for the amendment experiment, twelve antibiotics were detected (Table 2). The highest concentration was measured for doxycycline, DOX, (3605 µg kg^−1^), followed by lincomycin, LIN, (1196 µg kg^−1^) and tiamulin, TIA, (369 µg kg^−1^). Oxytetracycline (OTC) was the fourth most abundant antibiotic, with a mean value of 46 µg kg^−1^ (Figure 1). Other tetracyclines, sulfonamides, and quinolones were always lower than 40 µg kg^−1^ (Table 2). In soil, no antibiotic residues were found before amendment (t0), whereas, at t1, the mean concentrations of DOX, LIN, and TIA were 1095, 321, and 295 µg kg^−1^ dw, respectively (Table 3). The levels of all other detected antibiotics were lower than 50 µg kg^−1^ dw, and their concentrations were constant all along the experiment duration, except for OTC, which had a significant decrease from t1 to t15 (−25%, *p* = 0.006). The concentration trends of DOX and TIA over time were similar, with a significant decrease of about 60% from the initial concentration (t1) until the last time point (t15). In contrast, lincomycin decreased drastically (−70%) in just 14 days (t5), remaining lower than 20 µg kg^−1^ dw for the rest of the experiment (Figure 2; Table 3). Considering the sum of all antibiotics, a significant drop happened after two weeks (t5), and after two months (t7), the antibiotic sum was halved (from about 1700 to about 870 µg kg^−1^ dw).

### 2.3. ARGs in Manure and Soil

**Figure 3:**
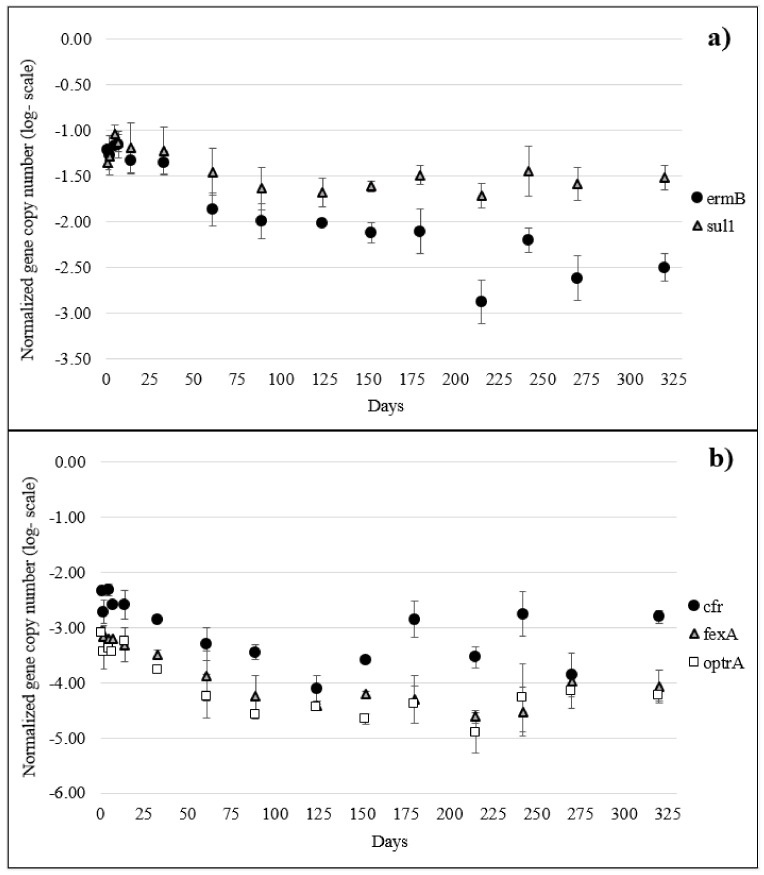
Relative abundances of *ermB*, *sul1* (**a**), *cfr*, *fexA*, and *optrA* (**b**). In both figures the copy gene number/copy number of 16S rRNA-log scale in soil during the incubation experiment are reported.

*Tet*M, *sul1*, and *ermB* were the most abundant ARGs in the slurry, accounting for −1.30, −1.52, and −1.66 log copy number/copy number of 16S rRNA, respectively. In the soil, before slurry spreading, no ARGs were detectable, while immediately after (t1) they all became detectable with the predominance of *sul1* and *ermB*; the other ARGs were always lower than −2 log (Table S8; Figure 3 and Figure S8). The concentration of *intI1* remained stable all along the experiment (from −1.9 to −1.35 log *intI1* copy number/copy number of 16S rRNA) as well as those of *tet*A, *tet*G, *tet*M, and *sul1*, for which no significant decline was observed from day 1 (t1) to day 320 (t15) (Figure 3a and Figure S8). On the contrary, the concentration of *ermB* (resistance to macrolides, lincosamides, and streptogramin B) remained quite stable through the first 61 days (t7), but it significantly dropped onward, and its concentration became lower than −2.0 log after 124 days (t9) (Figure 3a). Analogously, the relative abundances of *cfr*, *fexA*, and *optrA* decreased significantly starting from day 61 (t7) (Figure 3b). 

### 2.4. Spearman Correlations and Principal Component Analysis (PCA) Analysis in Soil

**Figure 4:**
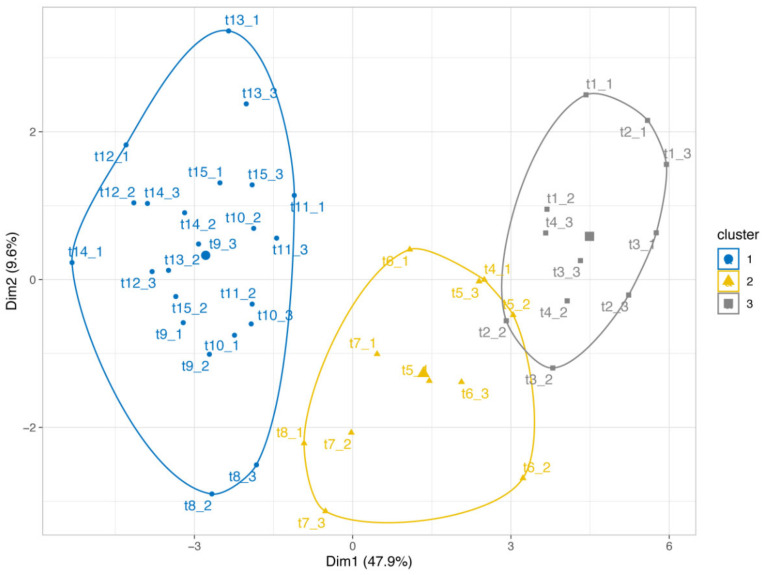
Principal Component Analysis Factor Map: Cluster 1 (●) grouped the observations relative to t9–t15 time points, Cluster 2 (▲) the observations relative to t5–t8 time points, and Cluster 3 (■) the observations relative to t1–t4 time points. Before the underscore is reported, the time point number (t1, t2, t3 … t15) and, after, the number of replicates (1, 2, 3).

The correlation matrix revealed that a large number of variables were positively correlated (Table S9). Among these, the five ARGs, *ermB*, *sul1*, *cfr*, *optrA*, and *fexA*, were all correlated with each other, as were the four antibiotics, LIN, DOX, TIA, and OTC. In addition, these five ARGs (except *sul1*) were also always correlated with LIN, DOX, TIA, and OTC. On the other hand, two significant negative correlations were observed, i.e., between C_mic_ and cumulative basal respiration (ΣCO_2_-C) and between C_mic_ and *tet*G. The PCA score plot (Figure 4) considering all the measured parameters gives a synthetic view of the incubation experiment evolution, identifying two axes that explain about 48% (Dimension 1) and 10% (Dimension 2) of variation, respectively. Cluster 3 includes the three observations for each time point of the first week since manure spreading (t1–t4). Later observations from t5 (14 days) to t8 (89 days) move along Dimension 1, and they group into Cluster 2, whereas all the subsequent observations were grouped in Cluster 1. The PC1 and PC2 were chosen to draw a biplot (Figure S3). PC1 was mainly driven by antibiotic residues of DOX, LIN, TIA, and OTC; by ARGs, *ermB*, *fexA*, *optrA*, and *cfr*; and by soil variables, pH, N_mic_, and CO_2_-C; whereas PC2 was driven by TN, TSN, and tetM.

Principal Component Analysis (PCA) was conducted, considering all the measured parameters, to provide a comprehensive view of the evolution of the incubation experiment. The results revealed the generation of three groups that clustered separately. Notably, 58.0% of the total variance among these groups was represented by the first two principal components (PCs), with PC1 and PC2 explaining 48.0% and 10.0% of the variance, respectively (refer to Figure 4). Specifically, the ‘first week’ group (t1–t4) could be differentiated from the ‘t5–t8’ and ‘t9–t15’ groups, demonstrating clear separation along the PC1 dimension. PC1 was primarily influenced by antibiotic residues such as DOX, LIN, TIA, and OTC, as well as by ARGs (*ermB*, *fexA*, *optrA*, and *cfr*) and soil variables including pH, Nmic, and CO_2_-C. On the other hand, PC2 was driven by TN, TSN, and *tet*M (Figure S9).

## 3. Discussion

The choice to perform a longitudinal mesocosm experiment was made to control for some of the large number of variables that may play a role in the studied phenomenon, such as environmental temperature, soil moisture, new uncontrolled spreading by the farmer, etc. In addition, knowledge of veterinary prescriptions in the nearly two years (650 days) preceding the experiment added complementary information to the results of the LC-HRMS analysis. Compared with other analytical techniques, LC-HRMS allows for greater selectivity with the definitive identification of analytes and the ability to simultaneously determine several drug classes. As is well known, quantitative analysis of antibiotics in complex matrices such as manure and soil is quite problematic, as pointed out by several researchers [3,12,13,14]. In particular, many different soil-adsorption mechanisms, such as hydrophobic interactions, hydrogen bonding, cation exchange, and complexation, affect their extraction from soil. Therefore, the validation data (Tables S4 and S5) could be considered satisfactory, except for the poor recoveries of three quinolones (difloxacin, marbofloxacin, and sarafloxacin) and one macrolide, spiramycin, in soil (<60%). However, taking into account the acceptable precisions (CV_wR_ ≤ 20%), these molecules were maintained among the searched compounds.

The decrease in soil pH immediately after fertilization at values lower than those of both pig slurry and unamended soil (Table 1) can be explained through the precipitation of calcium carbonate, as shown by mineral analysis (X-ray diffraction), which demonstrated an increase in calcite in the sand fraction of amended soil (Table S7 of Supplementary Material). The negative correlation between the amount of microbial community measured as C_mic_ and their activity measured as ΣCO_2_-C evolved during the basal respiration experiment should confirm the good adaptation of the microbial community hosted in the treated plots and be attributable to the great availability of energetic substrates supplied by pig slurry [15].

The twelve antibiotic residues found in pig slurry (Table 2) were among the most administered/authorized antibiotics in swine farming, except ciprofloxacin, which is the main metabolite of enrofloxacin, and sulfanilamide, whose presence could be due to the degradation of other sulfonamides [16] or Asulam herbicide [17]. It is worth noting that the four most abundant antibiotics in the slurry (DOX, LIN, TIA, and OXY) were all administered during the previous three months before the experiment started (Table 2). The study published by Berendsen et al. [11], who investigated the decline of various antibiotic families in pig manure, confirmed that these drugs are rather persistent, reaching 10% of their initial concentration (DT90) after 98 days, 892 days, 335 days, and 171 days, respectively. On the contrary, although the phenicol, florfenicol, was one of the most used drugs in the local swine farm (Figure S1) and, mainly, administered just two days before the experiment started, no residues of this molecule were detected. This observation was in agreement with the study of Nightingale et al. [18], who found that the half-life time of florfenicol in pig slurry was lower than two hours at neutral or basic pH, which was the case of the slurry spread in our experiment (pH = 7.20). Low concentrations of florfenicol amine (0.4–1.2 µg kg^−1^ dw, Table S8), the main metabolite of florfenicol, were detected in soil, confirming the recent prescription; florfenicol amine was undetectable in manure, probably because of its higher limit of detection in slurry than in soil (Tables S4 and S5 of Supplementary Material). Finally, as all the other prescribed drugs belonged to the beta-lactam and aminoglycoside classes (Figure S1), they could not be detected in either manure or soil. In the literature, it is well documented the rapid degradation of beta-lactam (penicillins and cephalosporins) after contact with manure [3], and for this reason, we did not investigate the presence of beta-lactams ARGs, representing a limit for the study. Among the most abundant antibiotics, lincomycin declined faster. This behavior was not related to a degradation process but, most probably, to a change in the sorption coefficient of this basic drug (pKa 7.6) due to the progressive increase in soil pH over the duration of the experiment. Wang and coauthors (2009) observed that an abrupt reduction in lincomycin sorption occurred just in the pH range from 7.0 to 7.5, which was explained by a decrease in the concentration of its cationic form in the solution. Since in our experiment the pH values of the soil just changed from 6.9 (t1) to 7.4 (t6), a similar explanation can be advanced. The choice of the eight monitored ARGs was carried out considering the data on local veterinary drug consumption. In 2019, sulfonamides (sulfadiazine and sulfadimethoxine), tetracyclines (doxycycline), lincomycin, macrolides (tylosin), tiamulin, and phenicols (florfenicol) were among the ten most administered antibiotic substances in Umbrian swine farming. Accordingly, all the measured ARGs encode for one or more of these compounds/classes. It is worth noting that *cfr* and *optrA* encode both for phenicols and for linezolid (Table S6), the first member of the class of oxazolidinone antibiotics primarily used as a last resort in humans to treat severe infections. Some studies have demonstrated that the extensive use of florfenicol in veterinary practices is associated with the production of linezolid-resistant genes [19,20]. Although *sul1* was one of the most abundant ARGs all along the experiment duration, its predominance cannot be explained by sulfonamide residues in soil, which were generally lower than 30 µg kg^−1^ dw. However, sulfonamides are widely used in Italian farming, and in 2019, they accounted for 15% of the total sales of veterinary antibiotics [21]. In particular, on the farm from which manure was collected, sulfadimethoxine was largely administered (in association with trimethoprim), but the last recorded administration was nine months before the start of the mesocosm experiment. On the other hand, sulfonamide resistance is widely diffused, as reported by the EFSA monitoring plan [22], even in antibiotic-free farming [23]. Indeed, *sul1*, an integron-associated gene, is considered the most common mobile resistant determinant in clinical settings and in the environment [24]. Another possible explanation for the predominance of *sul1* could be the presence in manure and, then, in soil of still-active transformation products of administered sulfonamides, resulting from animal metabolic reactions or biotic transformation from bacteria present in the manure biota. As well-known, sulfonamides are acetylated by animal metabolism, and although the acetylated forms are not active, they can be reversibly transformed by the parent drug acting as a sulfonamide reservoir. In addition, sulfadimethoxine can also degrade into still-active demethylated forms [25].

Before slurry application (t0), the relative abundances of ARGs in soil were below the method detection limit (about −5.3 log). Differently, in two similar experiments recently carried out in Flanders (Belgium), Van den Meersche et al. [9] and Huygens et al. [26] observed that the relative concentrations of nine ARGs (*tet*B, *tet*L, *tet*M, *tet*O, *tet*Q, *tet*W, *ermB*, *ermF*, *sul2*) were almost all measurable in soil already before fertilization [9,26]. In the same papers, the detection of various antibiotic residues already before the start of fertilization experiments was reported, too.

With regard to the several observed positive Spearman correlations among variables, it is worth noting that, after manure spread, there was an overall decline of several antibiotic residues and ARGs over time, and, therefore, most of these correlations could not necessarily indicate a cause-and-effect relationship. In fact, the majority of these correlations were among the four most abundant antibiotics (LIN, DOX, TIA, and OTC) and ARGs, *ermB*, *cfr*, *optrA*, and *fexA*. On the contrary, the “old” molecules, such as sulfadimethoxine, flumequine, and chlortetracycline, were much more sporadically correlated both with other antibiotic residues and with ARGs, probably because their levels were already stable when the slurry was collected at the beginning of the incubation study. It should be noted that “old” molecules have been used in agriculture for a long time. This long-term exposure to these antibiotics has caused an evolutionary adaptation of the gut bacteria. As a result, resistant determinants for “old” antibiotics, such as tetracyclines or sulfonamides, are very common in the gut flora, and the plasmids carrying these determinants are characterized by a low fitness cost. Thus, enteric bacteria carrying determinants for old molecules are often able to survive and thrive even in the absence of a selective pressure generated by antibiotics or their residues [27,28]. Positive correlations among *ermB*, *fexA*, and *optrA* could be explained by the detection of these genes in the same *Enterococcus* strains. Recently, *fexA* and *optrA* were found in *Enterococcocus feacalis* strains isolated from pig feces in Central Italy [20], and they are hosted on the same genetic cluster in *Enterococcus faecalis* isolates from humans and animals [29]. Yao and coauthors found that *ermB*, *fexA*, and *optrA* were located on the same plasmid in *Enterococcus gallinarum* of swine origin [30]. In addition, Kang et al. [31] detected *fexA*, *optrA*, and *ermA* in enterococci from swine farming facilities. This hypothesis cannot be confirmed using cultural methods, representing the limit of this study. A correlation between microbial biomass and some ARGs was also found. In particular, the N_mic_ was positively correlated with *fexA* and C_mic_ with *Ermb* and *TetG*. These results were probably due to an increase in microbial growth and ARGs concentrations after the pig manure spread. Competition between exogenous microorganisms coming from pig slurry and indigenous soil microbes can be hypothesized, leading to a shift in microbial community structure that was correlated with particular ARGs. In this regard, Yang and coauthors [32] reported that soil properties indirectly influenced ARGs by affecting bacterial diversity and were directly influenced by bacterial abundance, suggesting that ARGs are significantly associated with bacterial community structure. With regard to correlations between ARGs and antibiotic residues, unexplained results are frequently reported in the literature [8,9,26,33]. One of the problems is the impossibility of detecting exhaustively all the used parent drugs, their metabolites, and transformation products, which can be unknown and/or unavailable as reference standards [34]. For example, in our study, the lack of correlation between the three ARGs, *tet*A, *tet*G, and *tet*M, and tetracycline residues, which were predominant in both slurry and soil, was unclear. On the other hand, *cfr*, *fexA*, and *optrA* were correlated with several of the found drugs, although they do not encode resistance to any of these antibiotics. As before mentioned, in the local swine farm, the phenicol, florfenicol, was frequently administered (Figure S1), which can then explain the presence of these three ARGs, all encoding for this antibiotic class (Table S6). Unfortunately, *fexA* and *optrA* encode also for resistance to oxazolidinone antibiotics (particularly linezolid), and, therefore, the large use of florfenicol in livestock spreads these ARGs in the environment [11,35].

Cluster differentiation during the mesocosm experiment mainly occurs along PC1 (Figure 4). The vectors in Figure S9 (loading plot) illustrate that the dominant variables of this first component were DOX, LIN, *fexA*, *optrA*, *ermB*, and N_mic_, which decreased over time, and pH and C-CO_2_, which, on the contrary, increased over time. When these variables became quite stable (approximately after three months), the system evolution ended, as demonstrated by the overlapping of time points t9–t15 grouped in Cluster 1. The three ARGs of tetracyclines and the other physico-chemical parameters measured in soil play a minor role in the system’s evolution. This behavior agrees with the conclusions of similar recent studies [9,26] which sustain that antibiotic resistance is mostly introduced through fertilization with manure, as also demonstrated by the lack of correlations between *intI1* and ARGs.

## 4. Materials and Methods

### 4.1. Experimental Design and Sampling

In the present work, the presence and fate of a series of antibiotics and ARGs in soil after amendment with swine slurry were measured by measuring chemical, biological, and genetic parameters. For this purpose, a mesocosm experiment was designed by incubating pig slurry and agricultural soil, both collected from a local swine farm, for a period of 320 days. Soil samples were analysed before fertilization, immediately after fertilization (t1), and along fourteen time points (t2–t15). The pig slurry and soil were collected from a swine farm located in the municipality of Castiglione del Lago (Perugia, Central Italy). The pig slurry was collected in the farm lagoon, where the solid fraction comprising the slurry separated from the liquid one, which was periodically used as fertilizer in most of the farm’s fields cropped with sunflower, wheat, and corn. Veterinary drug prescriptions recorded by the farmer in the two years prior to the experiment (November 2017–September 2019) were acquired. During late summer 2019, an abundant amount of topsoil (Ap1 and Ap2 horizons, 0–10 cm) was collected from a farm’s crop field, never amended with pig slurry or other organic fertilizer. The soil had a clay loam texture (coarse sand 5.9%, fine sand 15.9%, silt 48.9%, clay 29.6%) and was classified as Eutric Cambisol (IUSS Working Group WRB, 2015). Once in the laboratory, the soil was sieved through a 4 mm-mesh. On September 11, 2019, about 50 L of slurry was collected from different points of the lagoon and immediately brought to the laboratory, where it was homogenized and analysed for pH (7.2), electric conductivity (1.6 mS cm^−1^) and nitrogen content (1.6 g L^−1^). On the same day, the mesocosm incubation experiment was set up. Six plastic boxes (41 cm × 33 cm) were filled with 8 kg of soil (bulk density 0.985 g cm^−3^, volume 8118 cm^3^) and brought to 50% of their water holding capacity. Based on the nitrogen content of the pig slurry and the soil volume in each box, three boxes were treated with 1.7 L of manure to assess a dosage of 200 kg N ha^−1^. The remaining three boxes were used as controls and were only supplemented with water. After manure/water addition, the contents of each box were thoroughly mixed to homogenate solid and liquid phases. The mesocosm incubation experiment was conducted indoors under temperature-controlled conditions (20 °C) [36]. For all the experiment duration, the soil humidity was maintained at 50% of its water holding capacity (determined following the method of ISO 11465, 1993). Chemical, biochemical, microbiological, and antibiotic residue analyses on treated soil samples were performed immediately before the experiment started (t0) and, after, at 15 time points from September 2019 to July 2020 (Table S1). Samples taken from each of the three boxes were analysed at each time point. With regard to control soil, chemical and biochemical analyses were performed at four time points (t1, t6, t12, t15).

### 4.2. Soil Analyses

Treated and control soil samples were periodically collected during the experimental period and analysed. The soil pH was determined potentiometrically in water (solid-liquid ratio: 1:2.5) after 30 min of stirring by a combined glass-calomel electrode (Hanna Instruments, Woonsocket, RI, USA). TOC and TN were determined by a dry combustion analyzer (EA-1110, Carlo Erba Instruments, Milan, Italy) after acid treatment (10% HCl solution) to dissolve inorganic carbon. The soluble forms of organic carbon (WSOC) and nitrogen (TSN) were extracted by a 0.5 M K_2_SO_4_ solution (solid:liquid ratio 1:4), shaken for 30 min, centrifuged at 1400× *g* for 10 min, and then filtered through Whatman 42 filter paper (Whatman, Kent, UK). The organic carbon and nitrogen in the filtered solution were determined by a TOC-500A (Shimadzu, Kyoto, Japan) analyzer after the addition of a few drops of concentrated H_3_PO_4_ to eliminate carbonates. The soil microbial biomass C (C_mic_) and N (N_mic_) were determined by the fumigation-extraction protocol [37], after 28 days of incubation at 25 °C and at 50% of soil water holding capacity. During the incubation, basal respiration was periodically measured by alkali absorption (1 M NaOH solution) of the developed carbon dioxide (CO_2_) and back-titration with a standardized HCl solution. The total amount of CO_2_ evolved during the incubation was expressed as the cumulative amount of CO_2_-C evolved during the experiment (ΣCO_2_-C). A semi-quantitative mineralogical analysis was carried out by X-ray diffraction, following the method reported in Agnelli et al. [38], on control and, at the end of the incubation, treated soil samples.

### 4.3. Antibiotic Analysis by LC-HRMS/MS

Reagents and materials—Merck KGaA (Darmstadt, Germany) supplied acetonitrile (ACN), methanol (MeOH), and acetic acid LC-MS grade. Formic acid was purchased from VWR Chemicals (Leuve, Belgium). EDTA sodium salt dihydrate and ammonium acetate were obtained from Sigma-Aldrich (St Louis, MO, USA). Deionized water was produced by the Milli-Q purification system (Millipore, Bedford, MA, USA). Strata X-C (200 mg, 6 mL) and Oasis HLB (200 mg, 6 mL) SPE columns were purchased from Phenomenex (Torrence, CA, USA) and Waters (Milford, MA, USA), respectively. Isolate NH2 columns were obtained from Biotage (Uppsala, Sweden).

Ciprofloxacin, difloxacin, enrofloxacin, flumequine, marbofloxacin, nalidixic acid, oxolinic acid, sarafloxacin, erythromycin A, anhydroerythromycin A, spiramycin I, tylosin A, tilmicosin, sulfadiazine, sulfaguanidine, sulfadimethoxine, sulfamerazine, sulfamethazine (sulfadimidine), sulfamethoxazole, sulfanilamide, sulfapyridine, sulfaquinoxaline, sulfathiazole, trimethoprim, chlortetracycline, doxycycline, methacycline, oxytetracycline, tetracycline, florfenicol, florfenicol amine, thiamphenicol, lincomycin, and tiamulin were obtained from Sigma-Aldrich. Sulfamonomethoxine was purchased from Dr. Ehrenstorfer (Augsburg, Germany); florfenicol-d3, 3-O-acetyltylosin, spiramycin I-d3, tylvalosin, 4-epichlortetracycline, 4-epitetracycline, 4-epioxytetracycline, and tulathromycin marker (CP60300) were purchased from TRC Inc. (Toronto, ON, Canada); sulfamethazine-13C6, sulfanilamide-13C6, and enrofloxacin-d5 were obtained from WITEGA (Berlin, Germany). Individual stock solutions of each analyte at 100 μg mL^−1^ were prepared as reported in Moretti et al. [39].

### 4.4. Determination of Antibiotics in Pig Slurry and Soil

Pig slurry—One gram and half of slurry were weighed in a 50 mL polypropylene tube, adding 50 µL of a solution containing each surrogate standard (enrofloxacin-d5, florfenicol-d3, methacycline, spiramycin I-d3, sulfamethazine-13C6, and sulfanilamide-13C6) at 1 μg mL^−1^, 0.39 g of Na_2_EDTA·2H_2_O, and 7 mL of ACN/H_2_O 80:20 (*v*/*v*) mixture containing 0.5% formic acid. Samples were shaken (20 min), sonicated (10 min) and centrifuged (1431× *g*, 10 min). The supernatant was transferred to a 15 mL polypropylene tube, and the solid residue was re-extracted with 3 mL of an ACN/H_2_O 80:20, (*v*/*v*) mixture containing 1% NH_3_. Samples were shaken, sonicated, and centrifuged again. The supernatants were reunited, centrifuged (1431× *g*, 10 min), and filtrated through an isolated NH_2_ column (500 mg, 6 mL—Biotage, Uppsala, Sweden) after conditioning with 6 mL of ACN. Extracts were dried under the N2 stream at 40 °C and reconstituted with 1.5 mL of ammonium acetate 0.2 M. Samples were sonicated (5 min), centrifuged (12,879× *g*, 10 min), and then injected into the LC-HRMS system.

Soil—The method was that published by Sargenti et al. [40] in river sediments with slight modifications. Briefly, about 30–40 g of each sample were air-dried at room temperature and the water percentage determined. 0.5 g of soil was weighed in a 50 mL polypropylene tube, adding 125 µL of internal standard solution at 1 μg mL^−1^. Samples were extracted with three consecutive extractions, each with a different mixture. The reunited supernatants were adjusted to pH 3 by HCl 1 N and diluted with water prior to being purified with two consecutive SPE cartridges: Oasis HLB (200 mg, 6 mL—Waters, Milford, MA, USA) and Strata X-C (200 mg, 6 mL—Penomenex, Torrance, CA, USA). The eluates were dried, solubilized with 0.5 mL of ammonium acetate 0.2 M, and injected.

LC-HRMS/MS conditions—LC-HRMS/MS platform consisted of a Thermo Ultimate 3000 (Thermo Scientific, San Jose, CA, USA) liquid chromatograph coupled to a Q-Orbitrap high-resolution hybrid mass spectrometer (Thermo Scientific) equipped with H-ESI II source operating in positive mode. The instrumental conditions were the same as described in Moretti et al. [39]. Briefly, analytes were separated by means of a Poroshell 120 EC-C18 column (100 × 3.0 mm, 2.7 µm, Agilent Technologies, Palo Alto, CA, USA) coupled to a pre-column (2.1 × 5 mm, 2.7 µm, Agilent Technologies). The flow rate was set at 0.25 mL min^−1^, injection volume at 5 µL and the column temperature was 30 °C. The chromatographic gradient is reported in Table S2. The acquisition was achieved in full scan/dd-MS2. In full scan, the data were acquired at a resolution of 70,000 FWHM (*m*/*z* 200). Automatic Gain Charge (AGC) was set at 3 × 106 ions for a maximum injection time (IT) of 100 ms. The precursor ions were filtered by the quadrupole, which operates at an isolation window of *m*/*z* 1.0. A resolution of 35,000 FWHM (*m*/*z* 200) was used. The AGC target was set at 1 × 106 ions for a maximum IT of 100 ms. The selected precursor and fragment ions, as well as the retention times (RT), are listed in Table S3. Validation studies of methods for slurry and soil are detailed in Supplementary Material (text; Tables S4 and S5). Analytes were quantified by constructing matrix-matched curves in the range 10 µg kg^−1^–150 µg kg^−1^ and applying the least squares method (R^2^ > 0.99). Samples and matrix-matched standards were injected both, such as and ten-fold diluted with ammonium acetate 0.2 M, to quantify also slurry and manure samples containing more than 150 µg kg^−1^.

### 4.5. DNA Extraction and Quantification of ARGs Using qPCR

Eight antibiotic-resistance genes were determined: *ermB*, *sul1*, *cfr*, *fexA*, *optrA*, *tetA*, *tetG*, and *tetM*. For the tetracyclines, genes with different mechanisms of resistance were included: two efflux pump genes (*tetA* and *tetG*) and one gene encoding for ribosomal protection proteins (*tetM*), as detailed in Table S6. In addition, class 1 integrase *intI1*, an indicator of horizontal ARG transmission, was also determined. *IntI1* encodes for an integron-integrase gene that helps antibiotic resistance genes spread from cell to cell, and, therefore, it has been proposed as a good indicator of anthropogenic pollution. 16S rRNA genes were also measured. Bacterial DNA was extracted from 0.25 g of pig slurry and soil samples using the QIAamp Fast DNA Stool Kit (Qiagen, Hilden, Germany) and the QIAamp PowerSoil DNA Isolation Kit (Qiagen), respectively, according to the manufacturer’s specifications. The quality and concentration of the extracted DNA were evaluated using a BioPhotometer spectrophotometer (Eppendorf, Hamburg, Germany), and the DNA was stored at −20 °C until analysis. The abundance of the genes was measured by quantitative Real-Time PCR (qPCR), using plasmidic constructs carrying target ARGs as standard curves. Standard curves were constructed as follows: selected ARGs were amplified from positive bacteria strains by PCR. The antibiotic, genes, primer sequence, annealing temperature, product size, positive strains, and references are listed in Table 4. PCR amplicons of each gene were purified using the High Pure PCR Product Purification Kit (Roche, Basel, Switzerland) and cloned into pCRTM 2.1-TOPO^®^ vectors and transferred into One Shot^®^ chemically competent *E. coli* TOP10F’ cells from the TOPO^®^ TA Cloning^®^ Kit (Invitrogen, Waltham, MA, USA) following the manufacturer’s instructions. Positive transformants were selected on LB agar supplemented with 50 µg mL^−1^ ampicillin (Sigma-Aldrich) and 40 mg mL^−1^ X-gaL (Promega, Madison, WI, USA) following the manufacturer’s instructions. Positive transformants were also confirmed by PCR (primer sequence, and annealing temperature are described in Table 4). Plasmid DNA was extracted from the transformants using the QIAprep^®^ Spin Miniprep Kit (Qiagen), analyzed by restriction analysis to confirm the presence and correct orientation of the insert, sequenced, and used as a standard curve for quantification purposes. The standard curves were constructed using 10-fold serial dilution, with a range of 10–10^6^ gene copy numbers µL^−1^. Only calibration curves with an efficiency between 90 and 110% and a linearity (R^2^) > 0.985 were considered acceptable. The presence of inhibitory substances in DNA samples was assessed by comparing the threshold values of two consecutive (10-fold) diluted samples [41]. Total 16S rRNA gene and the abundance of ARGs were determined using SYBR^®^ Green technology, preparing a reaction mixture of 10 µL of SYBR^®^ Green PCR Master Mix (Life Technologies, Carlsbad, CA, USA), 1 µL of each primer (0.5 µM), 2 µL sample DNA (20 ng), and 6 µL of nuclease-free water (total volume: 20 µL). The reaction was carried out using a QuantStudio 7 Flex (Applied Biosystem, Waltham, MA, USA) using the following cycling steps: 50 °C for 2 min, 95 °C for 5 min, 40 cycles at 95 °C for 20 s, 55 °C for 30 s, and 72 °C for 30 s. The specificity of the reaction products was monitored by melting curve analysis (0.2 °C s^−1^ to 95 °C, with acquisition data every 2 s). At each time point, a soil sample from each of the three boxes was analyzed with a standard curve and a negative control. Appropriate dilution (tenfold) was carried out when treated soil samples were analysed. The relative abundances of the ARGs were calculated by dividing the abundance of each gene by the 16S rRNA gene abundance, which represents the total amount of bacteria in the sample. This normalization was carried out to account for differences in extraction efficiency and in total bacterial number. A threshold cycle (CT) value of 36 was set as the detection limit (about −5.3 log, or 5 × 10^−6^ gene copy number/16S rRNA copy number).

### 4.6. Statistical Analysis

Relative abundances of ARGs were log transformed. Since the data did not pass the normality test (Shapiro-Wilk), a non-parametric approach was applied. The Kruskal-Wallis’s test, followed by post-hoc Dunn’s multiple comparison, was used to evaluate the differences among the fifteen time points. Correlations among variables were assessed using the Spearman rank test. The level of significance was set to 5%. Statistical analyses were performed with Stata (Stata version 16.1, StataCorp LLC, College Station, TX, USA) and R (version 3.3.1, RCoreTeam, 2021).

## 5. Conclusions

Before the pig slurry amendment, none of the monitored ARGs were detectable in soil, but they immediately after all became measurable (from 10^−4^ to 10^−2^ copy gene number/16S rRNA copy number), showing that the pig slurry amendment directly adds resistance genes to soil. On the other hand, the selective pressure exerted by antibiotic residues on soil-dwelling bacteria did not seem to play a significant role, as the increase in ARG concentrations was limited in time, although all ARGs were still detectable throughout the whole duration of the experiment. In addition, the PCA analysis, which produced three well-separated clusters, would suggest that the soil-slurry system evolves over time. Several of the observed positive correlations between antibiotic residues and ARGs were presumably due to the simultaneous input of antibiotics and resistance genes into the soil amended with pig slurry and the subsequent decline over time of several of the monitored variables. Finally, the extensive and recent administration of florfenicol in the swine herd from which the manure was taken probably accounts for the introduction into the soil of *cfr* and *optrA*, two ARGs that code for linezolid resistance, representing a potential risk to public health. Further research is needed in order to better understand the dynamics of ARGs and the antibiotic residues after soil fertilization with pig manure.

## Figures and Tables

**Table 1:** Chemical and biochemical properties of treated and control soil at the different time points (mean ± standard deviation) ^a^.

Days from Manure Conditioning	Time Points	Treated Soil							
		pH	TOC (%)	TN (%)	WSOC (mg kg^−1^)	TSN (mg kg^−1^)	C_mic_ (mg kg^−1^)	N_mic_ (mg kg^−1^)	ΣC-CO_2_ (µg kg^−1^)
1	1	6.87 ± 0.12	1.58 ± 0.09	0.28 ± 0.02	17.8 ± 3.8	18.0 ± 2.9	147 ± 8	14 ± 5	301 ± 16
2	2	6.91 ± 0.11	1.58 ± 0.11	0.26 ± 0.04	18.4 ± 3.1	19.0 ± 1.2	133 ± 12	14 ± 3	311 ± 24
5	3	7.02 ± 0.08	1.53 ± 0.09	0.27 ± 0.03	16.6 ± 0.7	17.8 ± 1.5	117 ± 11	14 ± 2	370 ± 20
7	4	7.21 ± 0.08	1.49 ± 0.14	0.27 ± 0.04	17.3 ± 2.3	18.4 ± 0.9	114 ± 15	13 ± 2	303 ± 45
14	5	7.29 ± 0.04	1.50 ± 0.16	0.24 ± 0.01	16.1 ± 3.2	19.1 ± 1.9	105 ± 4	12 ± 3	352 ± 31
33	6	7.32 ± 0.08	1.50 ± 0.11	0.25 ± 0.01	15.8 ± 4.2	19.9 ± 1.1	103 ± 5	12 ± 4	446 ± 101
61	7	7.58 ± 0.04	1.53 ± 0.13	0.26 ± 0.01	16.4 ± 2.2	14.5 ± 3.0	133 ± 23	8.3 ± 1.0	433 ± 69
89	8	7.37 ± 0.03	1.47 ± 0.18	0.25 ± 0.01	15.4 ± 1.1	18.9 ± 0.4	125 ± 29	5.2 ± 2.0	377 ± 84
124	9	7.38 ± 0.05	1.43 ± 0.09	0.25 ± 0.01	17.2 ± 0.9	18.8 ± 0.6	60 ± 6	2.1 ± 0.7	NA ^b^
152	10	7.54 ± 0.05	1.44 ± 0.11	0.25 ± 0.02	18.9 ± 1.6	22.0 ± 0.4	73 ± 9	3.7 ± 1.2	467 ± 52
180	11	7.31 ± 0.11	1.50 ± 0.10	0.28 ± 0.01	17.1 ± 0.3	20.6 ± 0.8	77 ± 4	3.1 ± 0.9	448 ± 48
215	12	7.42 ± 0.02	1.48 ± 0.09	0.29 ± 0.04	16.2 ± 1.8	22.6 ± 4.1	62 ± 8	3.0 ± 0.6	597 ± 155
242	13	7.44 ± 0.07	1.52 ± 0.11	0.30 ± 0.03	20.5 ± 0.5	22.6 ± 0.9	58 ± 2	2.9 ± 1.0	524 ± 26
270	14	7.42 ± 0.03	1.48 ± 0.16	0.28 ± 0.01	15.9 ± 2.8	18.2 ± 2.3	64 ± 7	1.9 ± 0.9	655 ± 138
320	15	7.48 ± 0.01	1.52 ± 0.10	0.26 ± 0.02	17.4 ± 1.8	21.3 ± 0.1	45 ± 1	1.8 ± 0.4	621 ± 153
	**Time Points**	**Control soil ^c^**							
		**pH**	**TOC** **(%)**	**TN** **(%)**	**WSOC** **(mg kg^−1^)**	**TSN** **(mg kg^−1^)**	**C_mic_** **(mg kg^−1^)**	**N_mic_** **(mg kg^−1^)**	**ΣC-CO_2_** **(µg kg^−1^)**
-	1	7.90 ± 0.04	1.02 ± 0.02	0.19 ± 0.01	14.7 ± 1.8	5.37 ± 1.59	92 ± 5.3	1.2 ± 0.2	550 ± 27
-	6	8.02 ± 0.03	1.05 ± 0.06	0.19 ± 0.01	13.9 ± 1.7	3.94 ± 2.59	76 ± 5.2	2.5 ± 0.4	557 ± 35
-	12	7.91 ± 0.04	1.04 ± 0.04	0.19 ± 0.01	12.1 ± 1.0	3.95 ± 0.98	42 ± 5.8	1.2 ± 0.2	573 ± 65
-	15	8.07 ± 0.06	1.03 ± 0.01	0.21 ± 0.01	11.1 ± 1.1	4.37 ± 1.09	56 ± 4.2	2.8 ± 0.3	594 ± 44

TOC = total organic carbon, TN = total nitrogen; WSOC = water-soluble organic carbon; TSN = total soluble nitrogen, C_mic_ = microbial biomass carbon; N_mic_ = microbial biomass nitrogen; ΣC-CO_2_ = cumulative amount of CO_2_-C evolved during the basal respiration experiment; ^a^ Values at each time point are the mean of the three measures carried out in each box prepared for the longitudinal experiment (treated and control soil); ^b^ Data not available due to a technical problem; ^c^ Analysis of control soil was carried out at t1 (experiment start), t6 (after 33 days), t12 (after 215 days), and at the end of the experiment (t15).

**Table 2:** Concentration, DT50/DT90, and time passed since the last administration of antibiotic residues found in the pig slurry used for the incubation experiment ^a^.

Antibiotic Class	Analyte	Concentration ± SD ^b^ (µg kg^−1^)	DT50/DT90 ^c^ (Days)	Time Passed Since the Last Administration ^d^ (Days)
Sulfonamides	Sulfadimethoxine (SDM)	5 ± 1	3.2/21	278
	Sulfamethazine (SMT)	4 ± 1	1.8/16	>650 ^e^
	Sulfanilamide (SNA)	29 ± 6	-	-
Tetracyclines	Doxycycline (DOX)	3605 ± 106	10/98	28
	Oxytetracycline (OTC) and epimer	46 ± 5	16/171	42
	Chlortetracycline (CTC) and epimer	20 ± 2	19/62	>650 ^e^
	Tetracycline (TC) and epimer	3 ± 1	12/111	>650 ^e^
Quinolones	Enrofloxacin (ENR)	13 ± 3	6/83	>650 ^e^
	Flumequine (FLU)	9 ± 2	44/146	>650 ^e^
	Ciprofloxacin (CIP)	3 ± 1	6/85	-
Lincosamides	Lincomycin (LIN)	1196 ± 24	269/892	23
Pleuromutilins	Tiamulin (TIA)	369 ± 17	101/335	65

^a^ Swine slurry has been collected and analysed immediately prior to the start of the incubation experiment; ^b^ Mean of three measurements (one for each box) ± standard deviation; ^c^ Dissipation times at 50% (DT50) and 90% (DT90) in pig manure are from Berendsen et al. [11]; ^d^ Supplied by the farmer (recorded administrations of the last 650 days); ^e^ No prescriptions were recorded for these drugs; however, chlortetracycline, tetracycline, enrofloxacin, and flumequine are authorized in swine farming.

**Table 3:** Concentrations (µg kg^−1^ dw) ^a^ of antibiotics in soil at the fifteen time points—continued.

Time Point	Day	FLOA ^b^	LIN	TIA	DOX	OTC ^c^	CTC ^c^	TC ^c^	ENR	FLU	CIP	SNA	SDM	SMT	TMP ^e^
1	1	0.4 ± 0.1	321 ± 42	295 ± 59	1095 ± 175	31 ± 5.8	9.1 ± 3.7	1.4 ± 0.5	8.8 ± 2.5	3.1 ± 0.6	1.9 ± 1.3	19 ± 2.0	5.0 ± 0.8	0.4 ± 0.1	0.2 ± 0.0
2	2	1.0 ± 0.1	329 ± 25	343 ± 61	1212 ± 289	49 ± 10	14 ± 3.5	1.4 ± 0.5	9.7 ± 2.0	4.0 ± 0.8	1.8 ± 0.8	26 ± 2.6	7.1 ± 1.4	0.6 ± 0.1	1.1 ± 0.4
3	5	1.0 ± 0.1	269 ± 37	235 ± 64	953 ± 159	41 ± 8.3	11 ± 2.3	1.4 ± 0.5	9.0 ± 1.7	3.4 ± 0.7	1.6 ± 0.5	27 ± 4.0	6.1 ± 1.2	0.4 ± 0.1	1.0 ± 0.0
4	7	1.0 ± 0.1	269 ± 53	297 ± 91	985 ± 384	39 ± 15	8.5 ± 3.5	1.0 ± 0.2	9.7 ± 2.9	3.0 ± 1.4	2.2 ± 0.0	24 ± 3.1	4.4 ± 1.4	0.3 ± 0.1	0.7 ± 0.1
5	14	1.2 ± 0.1	93 ± 38	176 ± 56	669 ± 81	30 ± 4.7	8.4 ± 1.2	0.9 ± 0.2	6.0 ± 0.9	2.3 ± 0.3	0.9 ± 0.2	31 ± 4.1	4.4 ± 0.9	0.3 ± 0.1	1.3 ± 0.1
6	33	1.5 ± 0.5	15 ± 6.4	202 ± 13	721 ± 64	38 ± 5.7	11 ± 1.4	1.5 ± 0.7	10 ± 0.8	4.5 ± 0.7	2.5 ± 0.3	26 ± 1.8	6.5 ± 0.7	0.5 ± 0.0	0.5 ± 0.0
7	61	0.7 ± 0.1	3.5 ± 0.7 ^d^	153 ± 11	484 ± 30	18 ± 0.7	11 ± 0.1	0.6 ± 0.1	7.5 ± 0.3	2.0 ± 0.0	1.7 ± 0.3	23 ± 6.7	3.0 ± 0.0	0.2 ± 0.0	0.1 ± 0.0
8	89	0.8 ± 0.3	5.5 ± 3.5	107 ± 20	491± 61	22 ± 0.7	7.0 ± 0.0	1.0 ± 0.0	6.6 ± 0.6	3.0 ± 0.0	2.1 ± 1.3	18 ± 5.2	4.0 ± 0.0	0.3 ± 0.0	0.5 ± 0.0
9	124	0.7 ± 0.1	5.0 ± 2.8	144 ± 1.4	543 ± 17	21 ± 0.7	7.9 ± 0.3	0.7 ± 0.1	8.8 ± 1.8	3.0 ± 0.0	1.5 ± 0.5	23 ± 4.5	4.0 ± 0.0	0.3 ± 0.1	0.2 ± 0.1
10	152	0.7 ± 0.1	4.5 ± 0.7	189 ± 19	689 ± 47	24 ± 4.2	8.4 ± 1.1	1.0 ± 0.0	11 ± 1.7	3.5 ± 0.7	2.0 ± 0.1	28 ± 2.7	4.5 ± 0.7	0.4 ± 0.1	0.2 ± 0.0
11	180	0.7 ± 0.1	3.5 ± 0.7	177 ± 11	620 ± 152	18 ± 1.4	7.9 ± 2.3	0.9 ± 0.1	9.6 ± 1.2	3.5 ± 0.7	1.5 ± 0.0	27 ± 5.7	4.5 ± 0.7	0.4 ± 0.1	0.3 ± 0.1
12	215	0.7 ± 0.1	4.0 ± 0.0	152 ± 22	442 ± 44	13 ± 1.4	6.6 ± 1.6	0.6 ± 0.1	7.8 ± 2.0	2.5 ± 0.7	1.2 ± 0.2	24 ± 1.5	4.0 ± 1.4	0.4 ± 0.1	0.2 ± 0.0
13	242	0.6 ± 0.1	3.5 ± 0.7	120 ± 36	499 ± 79	18 ± 5.7	6.8 ± 2.2	0.8 ± 0.3	7.3 ± 2.3	2.5 ± 0.7	1.1 ± 0.3	23 ± 0.5	4.0 ± 1.4	0.3 ± 0.1	0.3 ± 0.1
14	270	0.4 ± 0.1	3.0 ± 0.0	93 ± 7.8	418 ± 36	18 ± 4.9	5.5 ± 0.5	0.5 ± 0.1	6.8 ± 0.8	2.0 ± 0.0	2.1 ± 1.3	19 ± 4.9	3.0 ± 0.0	0.3 ± 0.1	0.1 ± 0.0
15	320	0.8 ± 0.1	2.9 ± 0.6	121 ± 29	408 ± 73	23 ± 4.6	8.3 ± 1.0	1.0 ± 0.1	5.6 ± 1.4	2.8 ± 0.5	0.8 ± 0.3	16 ± 3.2	4.8 ± 0.7	0.3 ± 0.0	0.2 ± 0.0

^a^ Mean of three determinations ± standard deviation (SD); ^b^ FLOA: florfenicol-amine; ^c^ Sum of parent drug and epimer; ^d^ Starting from time point 6 (t6), LIN did not decline significantly; ^e^ TMP: trimetoprim.

**Table 4:** Quantitative PCR primers and constructs.

Antibiotic	PCR Target	Primer Sequence (5′–3′)	Annealing Temperature (°C)	Product (bp)	Strain	Insert (µg/mL)	Plasmid	Construct [µg/mL]	Source
MLSB	*ermB*	CCGTGCGTCTGACATCTATCT	57/55	189	*E. coli R4287*	26	pCRTM 2.1-TOPO^®^	479	Guo et al. [42]
		GTGGTATGGCGGGTAAGTTTT							
Sulfonamides	*sul1*	CGCACCGGAAACATCGCTGCAC	56/55	163	*E. coli R4276*	147	pCRTM 2.1-TOPO^®^	425	Pei et al. [43]
		TGAAGTTCCGCCGCAAGGCTCG							
/	*intl1*	GGCTTCGTGATGCCTGCTT	55/55	146	*E. coli R4730*	69	pCRTM 2.1-TOPO^®^	455	He et al. [44]
		CATTCCTGGCCGTGGTTCT							
Chloramphenicol	*cfr*	GTTGGGAGTCATTTTGTATATC	55/55	179	*E. faecium V375*	51	pCRTM 2.1-TOPO^®^	370	This work
		CTTCWCCCATTCCCATAAAAG							
Florfenicol	*fexA*	ATTCTCCCGCAAATAACG	52/55	156	*E. faecalis V307*	71	pCRTM 2.1-TOPO^®^	793	Li et al. [45]
		TCGGCTCAGTAGCATCACG							
Oxazolidinone	*optrA*	GCTATTGTTGGTAGAAATGG	55/55	160	*E. faecalis V307*	51	pCRTM 2.1-TOPO^®^	507	This work
		CTTTCATCTTCAAAAGGCATC							
Tetracycline	*tetA*	GCTTCATGAGCGCCTGTTT	60/55	706	*E. coli R4730*	51	pCRTM 2.1-TOPO^®^	759	Pholwat et al. [46]
		CACCCGTTCCACGTTGTTAT							
	*tetG*	GCTCGGTGGTATCTCTGCTC	55/55	468	*S. typhimurim DT104*	69	pCRTM 2.1-TOPO^®^	645	Ng et al. [47]
		AGCAACAGAATCGGGAACAC							
	*tetM*	GTGGACAAAGGTACAACGAG	55/55	406	*S. delphini 2567*	112	pCRTM 2.1-TOPO^®^	378	Ng et al. [47]
		CGGTAAAGTTCGTCACACAC							
/	16s rRNA	ACTCCTACGGGAGGCAG	60/55	473	*E. coli R4829*	75	pCRTM 2.1-TOPO^®^	508	Van den Meersche et al. [41]
		ATTACCGCGGCTGCTGG							

## Data Availability

The original contributions presented in the study are included in the article and Supplementary Materials, further inquiries can be directed to the corresponding author.

## Supplementary Materials

The following supporting information can be downloaded at: https://www.mdpi.com/article/10.3390/antibiotics13060486/s1. Figure S1: Prescriptions of antibiotics (November 2017–September 2019) recorded at the swine farm in which manure was sampled (“others”: ampicillin, cefquinome, dicloxacillin, oxytetracycline, and spectinomycin); Figure S2: Extracted ion chromatograms: standard mixture of sulfonamides (100 ng mL^−1^); Figure S3: Extracted ion chromatograms: standard mixture of phenicols (100 ng mL^−1^); Figure S4: Extracted ion chromatograms: standard mixture of lincomycin, trimethoprim, and tiamulin (100 ng mL^−1^); Figure S5: Extracted ion chromatograms: standard mixture of macrolides (100 ng mL^−1^); Figure S6: Extracted ion chromatograms: standard mixture of quinolones (100 ng mL^−1^); Figure S7: Extracted ion chromatograms: standard mixture of tetracyclines (100 ng mL^−1^); Figure S8: Relative abundances of *tet*A, *tet*G, and *tet*M (copy gene number/copy number of 16S rRNA-log scale) in soil during the incubation experiment; Figure S9: Loading plot of PCA; Table S1: Sampling points; Table S2: LC gradient and mobile phases; Table S3: MS acquisition parameters for the analysis of antibiotics; Table S4: Summarised validation data for antibiotics in manure (spiked concentrations: 10, 50, and 100 μg kg^−1^); Table S5: Summarised validation data for antibiotics in soil (spiked concentrations: 1, 10, 50, and 100 μg kg^−1^ dw); Table S6: Mechanism of action of target genes; Table S7: Qualitative mineral composition of whole soil and sand, loam, and clay fractions of treated and control (CTR) samples; Table S8: Relative abundances (log) per time point of the ARGs in pig slurry and in the amended soil (mean of 3 measures ± SD). The abundance of each gene is divided by the abundance of the 16S rRNA gene copies present in the same sample for normalization. Table S9: Spearman correlation matrix (coefficients with a star highlight significant correlation, *p* < 0.05). References [3,11,21,48,49,50,51].
