# Supplementary material for: Impact of Soil Fertilization with Pig Slurry on Antibiotic Residues and Resistance Genes: A Longitudinal Study
Source: Antibiotics (Basel). 2024 May 24;13(6):486. doi: 10.3390/antibiotics13060486 (PMC11200711; doi:10.3390/antibiotics13060486)
Supplement: Supplementary file 1 [file antibiotics-13-00486-s001.zip › antibiotics-2991215-supplementary.pdf]

Supplementary Material

Impact of Soil Fertilization with Pig Slurry on Antibiotic Residues and Resistance Genes: A Longitudinal Study

Luisa Massaccesi [1], Elisa Albini [2], Francesca Romana Massacci [2,*], Danilo Giusepponi [2], Fabiola Paoletti [2], Stefano Sdogati [2], Francesco Morena [3], Alberto Agnelli [4], Angelo Leccese [4], Chiara Francesca Magistrali [2,5] and Roberta Galarini [2]

[1] National Research Council of Italy, Institute for Agriculture and Forestry Systems in the Mediterranean (ISAFOM-CNR), 06128 Perugia, Italy; luisa.massaccesi@cnr.it
[2] Istituto Zooprofilattico Sperimentale dell'Umbria e delle Marche "Togo Rosati", 06126 Perugia, Italy; e.albini@izsum.it (E.A.); d.giusepponi@izsum.it (D.G.); f.paoletti@izsum.it (F.P.); stefano.sdogati@izsum.it (S.S.); c.magistrali@izsler.it (C.F.M.); r.galarini@izsum.it (R.G.)
[3] Department of Chemistry, Biology and Biotechnology, Biochemical and Biotechnological Sciences, University of Perugia, 06122 Perugia, Italy; francesco.morena@unipg.it
[4] Department of Agricultural, Food and Environmental Science, University of Perugia, 06124 Perugia, Italy; alberto.agnelli@unipg.it (A.A.); angelo.leccese@unipg.it (A.L.)
[5] Istituto Zooprofilattico Sperimentale della Lombardia e dell'Emilia Romagna "Bruno Ubertini", 25124 Brescia, Italy
* Correspondence: fr.massacci@izsum.it; Tel.: +39-075-343074

Text: Validation of the LC-MS/MS method

Prior to method validation, the choice of analytes to be included in the method scope was investigated.

Some authors reported a rapid degradation of beta-lactams in animal manure, and accordingly, they excluded this class from the analytical protocol [1,2]. On the other hand, other researchers did not mention any degradation of beta-lactams, including some of them in their developed methods [3,4]. Therefore, a preliminary experiment was carried out, preparing two sets of four fortified pig slurry samples (100 μg kg^{-1}) adding 64 antibiotics belonging to eight families: beta-lactams, lincosamides, macrolides, phenicols, pleuromutilins, quinolones, sulphonamides and tetracyclines. The first set was carried out spiking manure just 15 minutes before extraction; the second one was prepared spiking manure 16 hours before analysis (overnight). The results are summarized in Figure S1. Accordingly, beta-lactams were not included in the developed analytical methods.

For slurry, the validation study was carried out by performing four replicates on three different occasions at 10, 50 and 100 μg kg^{-1}. Recoveries (> 70 %) and intra-lab reproducibility (CV_{wR} < 20%) were satisfactory for the majority of analytes (Table S4). The limits of detection (LODs) and quantification (LOQs) were from 0.1 to 6 μg kg^{-1} and from 0.3 to 20 μg μg kg^{-1}, respectively. In soil, the validation study was carried out at four concentrations (1, 10, 50 e 100 μg kg^{-1} dw) in three different days (Table S5). Recoveries were satisfactory, except for marbofloxacin (< 40 %), but since precision data for this analyte were still acceptable (CV_{wR} < 25%) it was included in the final protocol. LODs and LOQs ranged from 0.1 to 1.0 μg kg^{-1} dw and from 0.6 to 3.4 μg kg^{-1} dw, respectively.

Table S1. Sampling points.

Sampling Data	Time Points	Days from Manure Conditioning
11/09/2019	t0	0
12/09/2019	t1	1
13/09/2019	t2	2
16/09/2019	t3	5
17/09/2019	t4	7
25/09/2019	t5	14
14/09/2019	t6	33
11/11/2019	t7	61
11/12/2019	t8	89
13/01/2020	t9	124
10/02/2020	t10	152
09/03/2020	t11	180
14/04/2020	t12	215
11/05/2020	t13	242
08/06/2020	t14	270
28/07/2020	t15	320

Table S2. LC gradient and mobile phases.

Time (min)	% Mobile phase A (HCOOH 0.1% in H_2O)	% Mobile phase B (MeOH)
0	95	5
1	95	5
20	5	95
25	5	95
26	95	5
30	95	5

Table S3. MS acquisition parameters for the analysis of antibiotics.

N°	Analyte	RT (min)	RRT	Adduct	Adduct exact mass (*m/z*)	Ion 1	Ion 2	(N)CE
1	Sulfaguanidine	3.20	0.89	$[M+H]^+$	215.0597	156.0	108.1	35
2	Sulfanilamide	3.60	1.00	$[M+H]^+$	156.0114	92.1	108.1	40
	Sulfanilamide-$^{13}C_6$ (IS)[a]	3.60	-	$[M+H]^+$	162.0315	-	-	40
3	Florfenicolamine	3.60	0.35	$[M+H]^+$	248.0751	230.1	130.1	38
4	Sulfadiazine	6.70	0.74	$[M+H]^+$	251.0597	156.0	108.1	35
5	Sulfathiazole	7.30	0.81	$[M+H]^+$	256.0209	156.0	108.1	35
6	Sulfapyridine	7.75	0.86	$[M+H]^+$	250.0645	156.0	184.1	35
7	Sulfamerazine	7.95	0.88	$[M+H]^+$	265.0754	156.0	190.0	35
8	Lincomycin	8.50	0.71	$[M+H]^+$	407.2210	407.2	126.1	25
9	Trimethoprim	8.65	0.96	$[M+H]^+$	291.1451	230.1	261.1	50
10	CP 60300	8.70	0.73	$[M+2H]^{++}$	289.2066	289.2	420.3	18
11	Thiamphenicol	8.70	0.84	$[M+H]^+$	356.0121	308.0	338.0	25
12	4-epitetracycline	8.70	0.91	$[M+H]^+$	445.1605	267.1	201.1	50
13	Marbofloxacin	8.75	0.89	$[M+H]^+$	363.1463	320.1	363.1	20
14	Sulfamethazine	9.05	1.00	$[M+H]^+$	279.0910	124.1	204.0	40
	Sulfamethazine-$^{13}C_6$ (IS)[a]	9.05	-	$[M+H]^+$	285.1112	-	-	40
15	4-epioxytetracycline	9.50	0.72	$[M+H]^+$	461.1555	201.1	337.1	45
16	Tetracycline	9.60	0.73	$[M+H]^+$	445.1605	269.1	241.1	50
17	Oxytetracycline	9.80	0.75	$[M+H]^+$	461.1555	201.1	337.1	45
18	Ciprofloxacin	9.80	0.99	$[M+H]^+$	332.1405	332.1	245.1	50
19	Enrofloxacin	9.85	1.00	$[M+H]^+$	360.1718	203.1	245.1	55
	Enrofloxacin-d_5 (IS)[a]	9.85	-	$[M+H]^+$	365.2032	-	-	55
20	Sulfamethoxazole	10.15	1.21	$[M+H]^+$	254.0594	156.0	254.0	30
21	Difloxacin	10.30	1.05	$[M+H]^+$	400.1467	400.1	356.2	33
22	Sulfamonomethoxine	10.40	1.15	$[M+H]^+$	281.0703	156.0	108.1	35

23	Florfenicol	10.40	1.00	$[M+H]^+$<br>$[M+NH_4]^+$	358.0077<br>375.0343	340.0	241.0<br>358.0	10
	Florfenicol-d_3 (IS)[a]	10.40	-	$[M+H]^+$	361.0266	-	-	10
24	Sarafloxacin	10.60	1.08	$[M+H]^+$	386.1311	299.1	386.1	60

N°	Analyte	RT (min)	RRT	Adduct	Adduct exact mass (*m/z*)	Ion 1	Ion 2	(N)CE
25	4-epichlortetracycline	11.00	0.84	$[M+H]^+$	479.1216	303.0	371.0	50
26	Chlortetracycline	11.90	0.90	$[M+H]^+$	479.1216	303.0	275.0	50
27	Spiramycin	11.90	1.00	$[M+2H]^{++}$	422.2643	422.3	540.3	10
	Spiramycin-I-d_3 (IS)[a]	11.90	-	$[M+2H]^{++}$	423.7737	-	-	10
28	Sulfadimethoxine	12.40	1.37	$[M+H]^+$	311.0809	156.0	108.1	38
29	Sulfaquinoxaline	12.85	1.42	$[M+H]^+$	301.0754	156.0	108.1	30
30	Oxolinic Acid	13.05	1.32	$[M+H]^+$	262.0710	160.0	234.1	75
	Metacycline (IS)[a]	13.15	-	$[M+H]^+$	443.1449	-	-	30
31	Tilmicosin	13.40	1.13	$[M+2H]^{++}$	435.2900	695.5	435.3	25
32	Doxycycline	13.55	0.90	$[M+H]^+$	445.1605	267.1	321.1	50
33	Nalidixic acid	14.80	1.49	$[M+H]^+$	233.0921	233.1	205.1	50
34	Tiamulin	15.10	1.27	$[M+H]^+$	494.3299	192.1	494.3	18
35	Flumequine	15.25	1.55	$[M+H]^+$	262.0874	238.1	262.1	70
36	Tylosin A	15.35	1.29	$[-oxane+2H]^{++}$	363.7248	174.1	336.2	22
37	Erythromycin A	15.60	1.31	$[M+H]^+$	734.4685	576.4	734.5	12
38	3-O-acetyltylosin	15.70	1.32	$[-oxane+2H]^{++}$	384.7301	109.1	174.1	22
39	Anhydroerythromycin	16.20	1.10	$[M+H]^+$	716.4580	558.4	158.2	12
40	Tylvalosin	17.40	1.46	$[-oxane+2H]^{++}$	426.7588	109.1	174.1	22

[a]Except for enrofloxacin (enrofloxacin-d_5), ciprofloxacin (enrofloxacin-d_5) and sulfanilamide (sulfanilamide-$^{13}C_6$) in soil (see Table 5), internal standard (IS) were used only to check recoveries and not to quantify the analytes

Table S4. Summarised validation data for antibiotics in manure (spiked concentrations: 10, 50 and 100 μg kg^{-1}).

**Analyte**	**Recovery (%)**	**CV_r[a] (%)**	**CV_{wR}[a] (%)**	**LOD (μg kg^{-1})**	**LOQ (μg kg^{-1})**	**Analyte**	**Recovery (%)**	**CV_r (%)**	**CV_{wR} (%)**	**LOD (μg kg^{-1})**	**LOQ (μg kg^{-1})**
3-O-Acetyltylosin	71	8.8	13	2.7	9.0	Sulfamerazine	85	5.4	11	0.2	0.7
CP 60300[b]	92	6.3	8.7	0.7	2.2	Sulfamethazine	84	5.0	7.4	0.2	0.5
Erythromycin A[c]	85	5.3	5.6	0.1	0.4	Sulfamethoxazole	86	7.6	10	0.3	1.0
Spiramycin	82	7.4	17	0.2	0.7	Sulfamonomethoxine	84	8.2	9.3	0.3	1.2
Tilmicosin	93	5.1	9.8	0.2	0.5	Sulfanilamide	*120*	*15*	*19*	10	30
Tylosin A	72	11	13	2.7	9.0	Sulfapyridine	82	5.9	9.2	0.2	0.8
Tylvalosin	67	8.5	15	3.5	12	Sulfaquinoxaline	83	5.6	6.4	0.2	0.7
Ciprofloxacin	89	6.3	11	0.7	2.3	Sulfathiazole	82	6.6	13	0.3	0.9
Difloxacin	94	11	11	0.3	1.0	Chlortetracycline[d]	78	7.2	9.7	0.3	0.9
Enrofloxacin	97	6.6	10	0.7	2.2	Doxycycline	81	6.0	10	4.1	14
Flumequine	96	6.5	9.9	0.2	0.8	Oxytetracycline[d]	81	7.5	10	0.2	0.8
Marbofloxacin	97	7.0	9.1	0.8	2.5	Tetracycline[d]	86	5.2	8.2	0.3	1.0
Nalidixic Acid	96	5.9	6.9	0.1	0.5	Florfenicol	97	9.4	9.4	3.1	10
Oxolinic Acid	96	5.9	7.2	0.1	0.3	Florfenicolamine	98	5.6	8.4	3.2	11
Sarafloxacin	90	9.5	13	0.3	1.1	Thiamphenicol	96	7.0	8.0	3.4	12
Sulfadiazine	85	5.9	9.7	0.2	0.7	Lincomycin	87	3.9	6.2	0.5	1.6
Sulfadimethoxine	82	7.8	10	0.4	1.2	Tiamulin	95	6.6	7.1	0.2	0.6
Sulfaguanidine	87	8.0	17	3.5	12	Trimethoprim	97	7.2	8.6	4.2	14

[a]CV_r and CV_{wR}: coefficients of variation obtained in repeatability and within laboratory reproducibility conditions, respectively; [b]Tulathromycin marker; [c]Sum of Erythromycin A and anhydroerythromycin A; [d]Sum of parent drug and its epimer;

Table S5. Summarised validation data for antibiotics in soil (spiked concentrations: 1, 10, 50 and 100 µg kg^{-1} dw).

Analyte	Recovery (%)	CV_r[a] (%)	CV_{wR}[a] (%)	LOD (µg kg^{-1} dw)	LOQ (µg kg^{-1} dw)	Analyte	Recovery (%)	CV_r (%)	CV_{wR} (%)	LOD (µg kg^{-1} dw)	LOQ (µg kg^{-1} dw)
3-O-Acetyltylosin	75	12	13	0.3	0.9	Sulfamerazine	84	9.7	11	0.2	0.6
CP 60,300[b]	58	10	12	0.3	1.0	Sulfamethazine	83	8.9	10	0.3	0.8
Erythromycin A[c]	68	9.9	11	0.2	0.8	Sulfamethoxazole	83	9.2	10	0.2	0.7
Spiramycin	38	16	17	0.7	2.3	Sulfamonomethoxine	82	8.2	10	0.2	0.7
Tilmicosin	64	12	13	0.3	0.9	Sulfanilamide[d]	103	11	11	0.3	1.2
Tylosin A	75	14	14	0.5	1.7	Sulfapyridine	83	10	11	0.2	0.8
Tylvalosin	67	17	17	0.3	0.9	Sulfaquinoxaline	77	8.9	10	0.3	0.8
Ciprofloxacin[d]	102	13	21	1.0	3.4	Sulfathiazole	80	12	13	0.3	1.1
Difloxacin	39	12	13	0.2	0.6	Chlortetracycline[e]	77	10	12	0.3	0.9
Enrofloxacin[d]	113	9.8	11	0.5	1.6	Doxycycline	74	17	20	0.6	2.1
Flumequine	83	8.3	8.7	0.1	0.5	Oxytetracycline[e]	72	8.3	8.9	0.2	0.7
Marbofloxacin	32	19	20	0.6	2.0	Tetracycline[e]	67	10	11	0.4	1.3
Nalidixic Acid	79	6.7	8.5	0.1	0.3	Florfenicol	91	9.3	11	0.4	1.5
Oxolinic Acid	70	9.8	11	0.1	0.5	Florfenicolamine	80	11	11	0.3	1.1
Sarafloxacin	51	9.6	10	0.4	1.2	Thiamphenicol	88	9.5	9.5	0.3	1.2
Sulfadiazine	86	10	11	0.2	0.8	Trimethoprim	90	8.4	11	0.2	0.7
Sulfadimethoxine	82	8.0	9.2	0.2	0.6	Lincomycin	66	15	18	0.5	1.7
Sulfaguanidine	84	16	19	0.7	2.3	Tiamulin	79	11	12	0.2	0.8

[a]CV_r and CV_{wR}: coefficients of variation obtained in repeatability and within laboratory reproducibility conditions, respectively; [b]Tulathromycin marker; [c]Sum of Erythromycin A and anhydroerythromycin; [d]Analytes quantified through internal standardization (isotopic dilution); [e]Sum of parent drug and its epimer;

Table S6. Mechanism of action of target genes [5].

Resistance	PCR target	Mechanism of action
Macrolide, lincosamides, streptogamin A. Macrolides are classified as "Critically Important" (the top category) by the World Health Organization (WHO,2018) [6]. Macrolides and lincosamides account for 6 % and 7 %, respectively, of all veterinary drugs sold in Italy in 2019 (EMA, 2021) [7]	*ermB*	Antibiotic target alteration
Sulfonamides. *Sul1* is one of the most studied resistance genes in environmental samples. This family is classified as "Highly Important" (the second category) by the World Health Organization (WHO, 2018) [6] it accounts for 15 % of all veterinary drugs sold in Italy in 2019 (EMA, 2021) [7]	*sul1*	Antibiotic target substitution
Phenicols (including florfenicol), lincosamides (including lincomycin), oxazolidinones, pleuromutilins (including tiamulin), streptogramin A	*cfr*	Antibiotic target alteration
Phenicols	*fexA*	Directed pumping of antibiotic out of a cell
Phenicols and oxazolidinones	*optrA*	Antibiotic target protection
Tetracyclines. The World Health Organization (WHO,2018) [6] classifies this family as "Highly Important" (the second category). Tetracyclines account for 27% of all veterinary drugs sold in Italy in 2019 (EMA, 2021) [7]	*tetA*	Directed pumping of antibiotic out of a cell
	tetG	Directed pumping of antibiotic out of a cell
	tetM	Antibiotic target protection

Table S7. Qualitative mineral composition of whole soil and sand, loam and clay fractions of treated and control (CTR) samples.

**Whole sample**						
	**Quartz**	**calcite**	**plagioclase**	**orthoclase**	**micas**	**kaolinite**
Treated soil	+++	+++	+(+)	-	(+)	traces
CTR	+++	++	++	-	(+)	Traces
**Fractions**						
	**Quartz**	**calcite**	**plagioclase**	**orthoclase**	**micas**	**kaolinite**
Sand	+++	+++++(+)	+(+)	-	Traces	-
Sand (CTR)	+++	++(+)	+++(+)	(+)	(+)	-
Loam	+++(+)	+++(+)	++(+)	-	(+)	Traces
Loam (CTR)	+++(+)	+++(+)	++(+)	-	(+)	Traces
Clay	(+)	-	-	-	(+)	-
Clay (CTR)	(+)	-	-	-	(+)	Traces

+: about 10%; (+): about 5%;

Table S8. Relative abundances (log) per time point of the ARGs in pig slurry and in the amended soil (mean of 3 measures ± SD). The abundance of each gene is divided by the abundance of the 16S rRNA gene copies present in the same sample for normalization.

Time Point[a]	IntI1	ErmB	Sul1	Cfr	FexA	OptrA	Tet(A)	Tet(G)	Tet(M)
Slurry	−1.92 ± 0.20	−1.66 ± 0.09	−1.52 ± 0.11	−2.00 ± 0.13	−3.05 ± 0.30	−3.00 ± 0.24	−4.00 ± 0.08	−3.5 ± 0.16	−1.30 ± 0.07
1	−1.74± 0.32	−1.22 ± 0.06	−1.36 ± 0.08	−2.33 ± 0.03	−3.06 ± 0.07	−3.10 ± 0.09	−2.46 ± 0.05	−3.28 ± 0.17	−2.06 ± 0.26
2	−1.60 ± 0.17	−1.28 ± 0.21	−1.29 ± 0.13	−2.71 ± 0.21	−3.17 ± 0.20	−3.45 ± 0.31	−2.29 ± 0.08	−3.44 ± 0.18	−2.67 ± 0.15
3	−1.35 ± 0.17	−1.17 ± 0.03	−1.05 ± 0.10	−2.32 ± 0.11	−3.20 ± 0.01	−3.38 ± 0.09	−2.03 ± 0.03	−3.05 ± 0.29	−3.06 ± 0.22
4	−1.44 ± 0.17	−1.16 ± 0.14	−1.14 ± 0.10	−2.57 ± 0.06	−3.21 ± 0.02	−3.46 ± 0.05	−2.11 ± 0.03	−3.21 ± 0.04	−2.86 ± 0.06
5	−1.61 ± 0.64	−1.34 ± 0.13	−1.20 ± 0.28	−2.58 ± 0.26	−3.31 ± 0.31	−3.26 ± 0.07	−1.84 ± 0.16	−2.50 ± 0.18	−2.82 ± 0.01
6	−1.50 ± 0.38	−1.36± 0.13	−1.22 ± 0.25	−2.86 ± 0.05	−3.49 ± 0.09	−3.78 ± 0.04	−2.30 ± 0.22	−2.83 ± 0.31	−2.69 ± 0.16
7	−1.67 ± 0.77	−1.87 ± 0.18	−1.46 ± 0.25	−3.30 ± 0.31	−3.88 ± 0.45	−4.25 ± 0.39	−2.26 ± 0.24	−2.84 ± 0.14	−3.02 ± 0.26
8	−1.74 ± 0.40	−2.00 ± 0.19	−1.64 ± 0.23	−3.45 ± 0.14	−4.24 ± 0.38	−4.58 ± 0.07	−2.55 ± 0.30	−3.41 ± 0.25	−3.55 ± 0.26
9	−1.76 ± 0.32	−2.02 ± 0.01	−1.69 ± 0.16	−4.11 ± 0.24	−4.42 ± 0.08	−4.44 ± 0.05	−2.52 ± 0.15	−2.72 ± 0.29	−2.63 ± 0.08
10	−1.80 ± 0.34	−2.13 ± 0.11	−1.62 ± 0.06	−3.59 ± 0.01	−4.20 ± 0.05	−4.66 ± 0.10	−2.55 ± 0.12	−3.01 ± 0.20	−2.71 ± 0.35
11	−1.57 ± 0.11	−2.11 ± 0.25	−1.49 ± 0.10	−2.85 ± 0.33	−4.30 ± 0.43	−4.39 ± 0.34	−2.33 ± 0.14	−2.82 ± 0.19	−2.98 ± 0.19
12	−1.69 ± 0.04	−2.88 ± 0.24	−1.71 ± 0.13	−3.54 ± 0.19	−4.62 ± 0.11	−4.90 ± 0.36	−2.40 ± 0.08	−2.63 ± 0.24	−3.21 ± 0.29
13	−1.58 ± 0.23	−2.21 ± 0.13	−1.45 ± 0.27	−2.76 ± 0.40	−4.52 ± 0.44	−4.28 ± 0.62	−2.31 ± 0.28	−2.45 ± 0.48	−2.58 ± 0.17
14	−1.52 ± 0.13	−2.62 ± 0.25	−1.60 ± 0.18	−3.85 ± 0.39	3.97 ± 0.19	−4.16 ± 0.31	−3.62 ± 0.24	−2.64 ± 0.12	−2.64 ± 0.21
15	−1.39 ± 0.15	−2.51 ± 0.15	−1.52 ± 0.14	−2.80 ± 0.11	−4.08 ± 0.30	−4.23 ± 0.10	−3.55 ± 0.16	−2.58 ± 0.12	−2.66 ± 0.16

[a]Before the amendment (T0) ARGs were not detectable

Table S9. Spearman correlation matrix in soil (coefficients with star highlight significant correlation, $p<0.05$)[a].

	Ermb	*Sul1*	*Cfr*	*Fexa*	*Optra*	*TetA*	*TetG*	C_{mic}	N_{mic}	C-CO_2	FLU	LIN	TIA	SDM	OTC	CTC	DOX	SUM
ermb	1																	
sul1	0.8107*	1																
cfr	0.7439*	0.7635*	1															
fexa	0.8528*	0.7727*	0.7273*	1														
optra	0.8285*	0.7781*	0.8172*	0.9105*	1													
TetA	0.6830*	0.8127*	0.5937*	0.4901	0.5235	1												
TetG	-0.4002	0.0456	-0.0979	-0.295	-0.1409	0.08	1											
C_{mic}	0.5897*	0.3359	0.2863	0.5374	0.3792	0.3466	-0.5786*	1										
N_{mic}	0.4496	0.3113	0.2869	0.5585*	0.4198	0.1719	-0.4084	0.7066*	1									
C-CO_2	-0.4021	-0.2164	-0.3014	-0.311	-0.2573	-0.2227	0.3867	-0.5615*	-0.3286	1								
FLU	0.131	0.0396	0.1179	0.0293	-0.0313	-0.0579	-0.2155	0.0946	0.1872	-0.0105	1							
LIN	0.7916*	0.5537*	0.5671*	0.7589*	0.6812*	0.5033	-0.4799	0.5073	0.5141	-0.2399	0.2081	1						
TIA	0.6553*	0.4651	0.5528*	0.6386*	0.5862*	0.4138	-0.347	0.4823	0.4838	-0.2269	0.3987	0.7863*	1					
SDM	0.3173	0.2785	0.4007	0.3066	0.2938	0.1053	-0.216	0.109	0.3501	-0.0179	0.7870*	0.4318	0.5304	1				
OTC	0.6869*	0.5155	0.5625*	0.6897*	0.6428*	0.2493	-0.432	0.3894	0.4944	-0.1539	0.5601*	0.7109*	0.6650*	0.7857*	1			
CTC	0.4138	0.3961	0.3056	0.3515	0.3164	0.2839	-0.2621	0.3861	0.4963	-0.0266	0.5762*	0.3628	0.5178	0.7306*	0.6952*	1		
DOX	0.8251*	0.5694*	0.6263*	0.7098*	0.6615*	0.4667	-0.5027	0.5032	0.4638	-0.2917	0.4299	0.8581*	0.8739*	0.5637*	0.7897*	0.5312	1	
SUM[b]	0.7998*	0.5502*	0.6150*	0.7111*	0.6663*	0.4658	-0.4835	0.485	0.4633	-0.2554	0.4104	0.8825*	0.9138*	0.5731*	0.7806*	0.5506*	0.9878*	1

[a]Due to the high number of variables, uncorrelated variables have been omitted as well as the positive correlation between soluble carbon (WSOC) and soluble nitrogen (TSN) and between enrofloxacin and flumequine; [b]Sum of all measured antibiotics

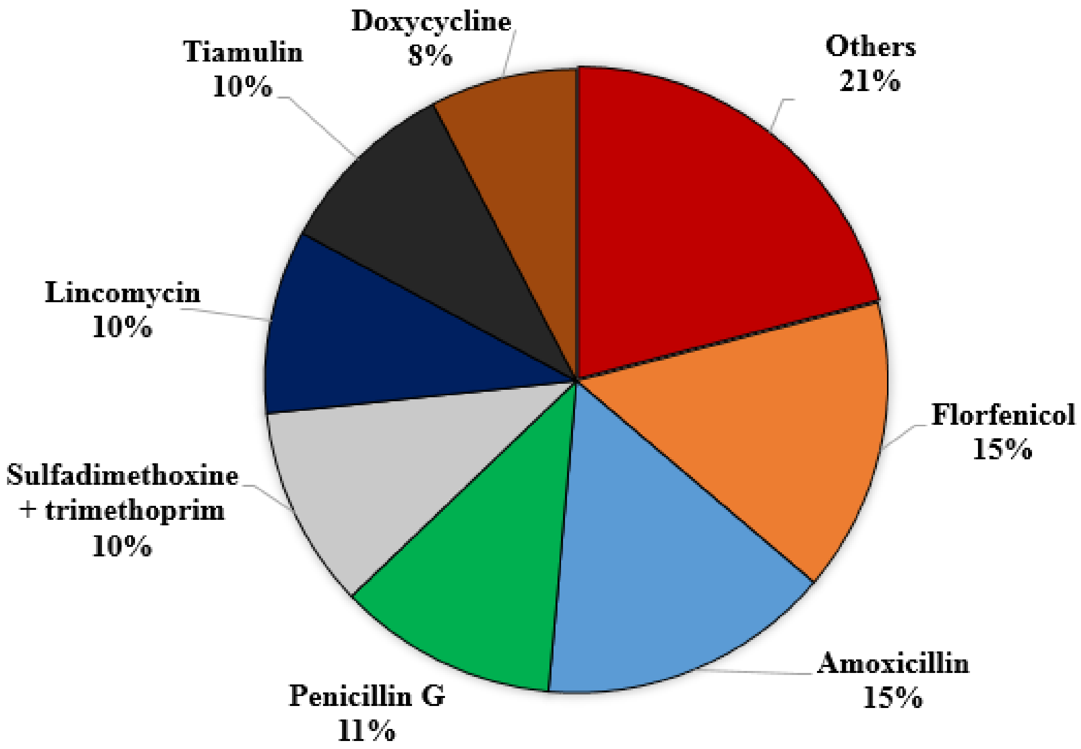

Figure S1. Prescriptions of antibiotics (November 2017 - September 2019) recorded at the swine farm in which manure was sampled ("others": ampicillin, cefquinome, dicloxacillin, oxytetracycline and spectinomycin).

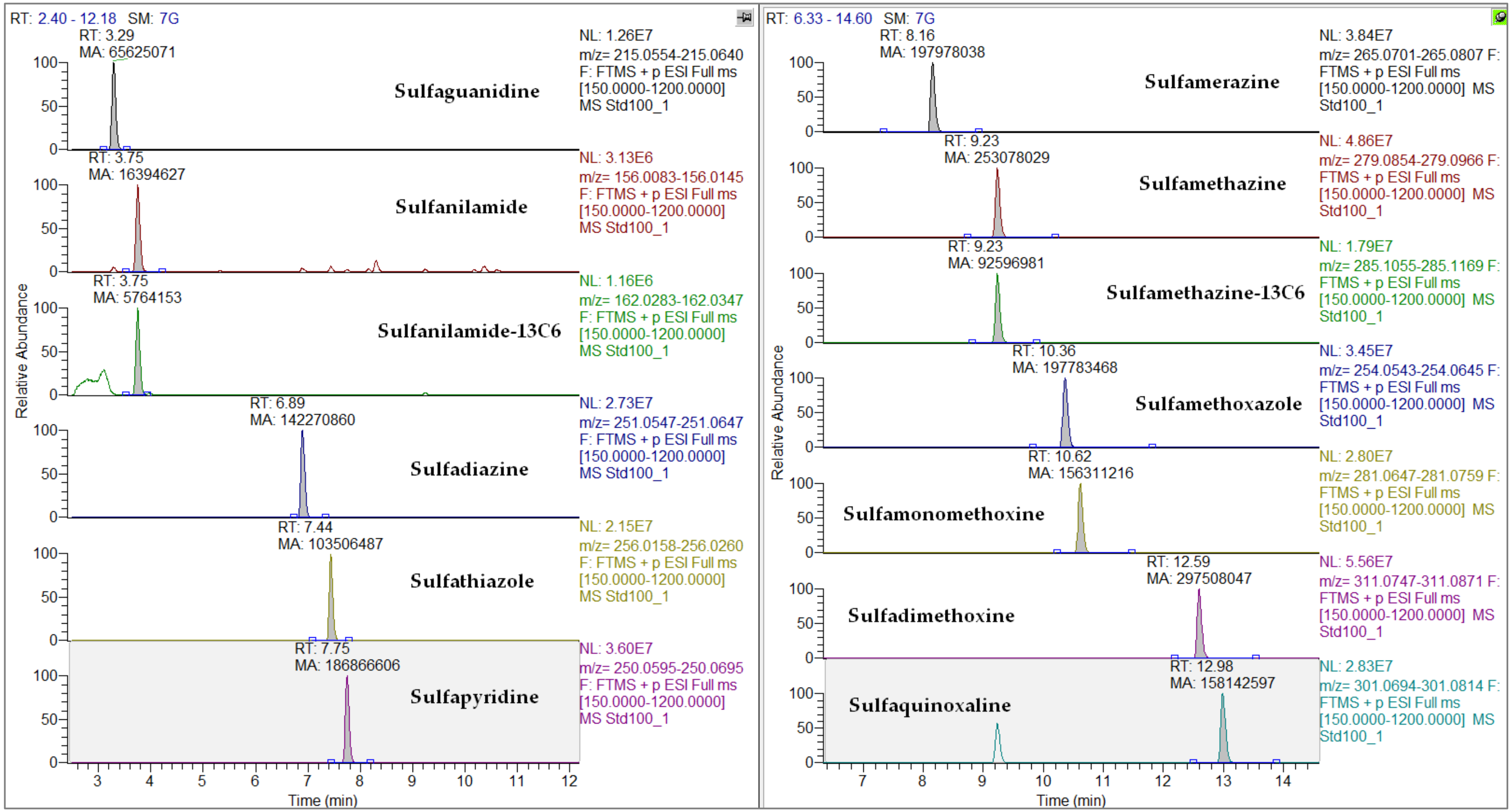

Figure S2. Extracted ion chromatograms: standard mixture of sulfonamides (100 ng mL^{-1}).

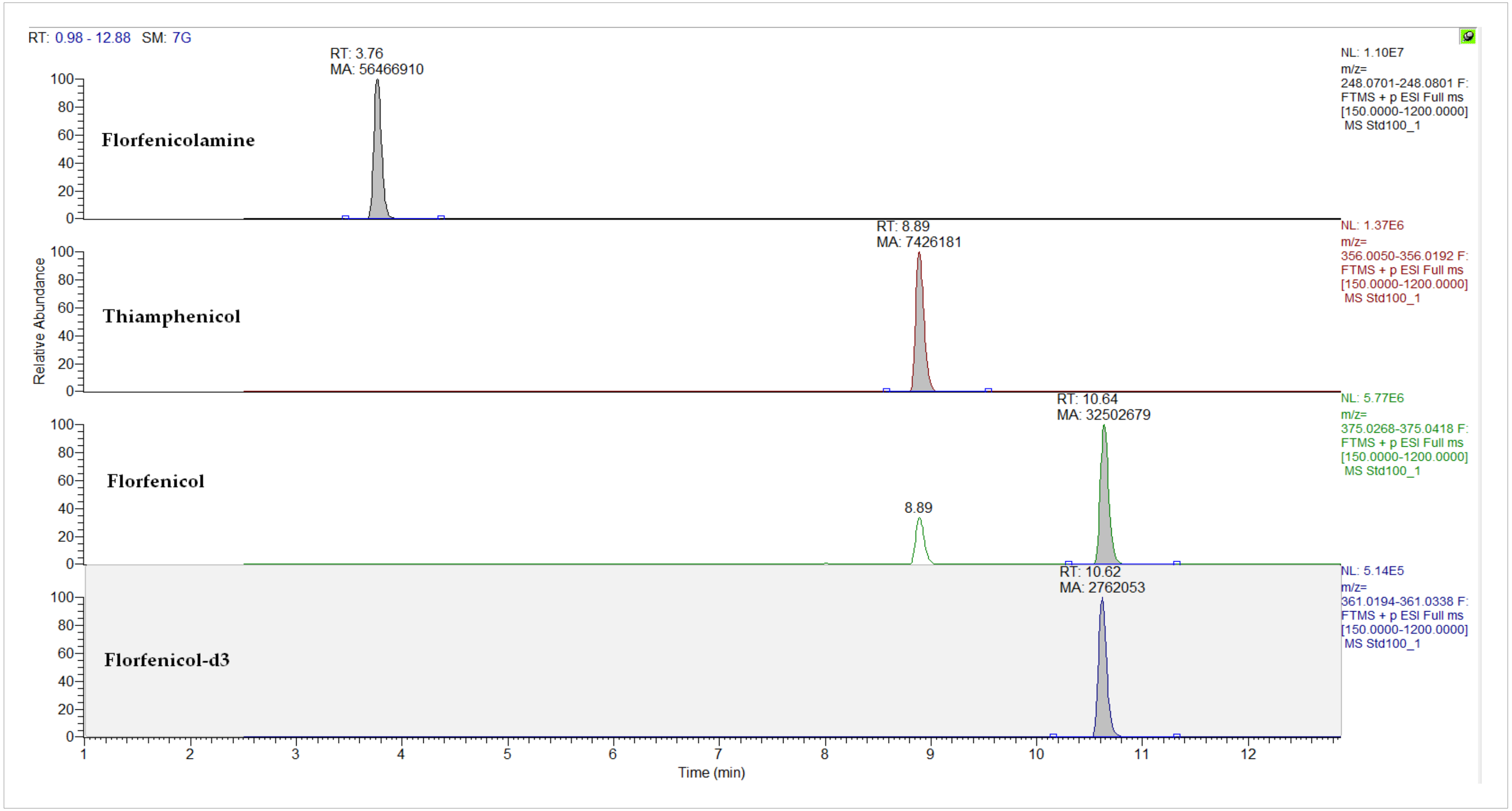

Figure S3. Extracted ion chromatograms: standard mixture of phenicols (100 ng mL^{-1}).

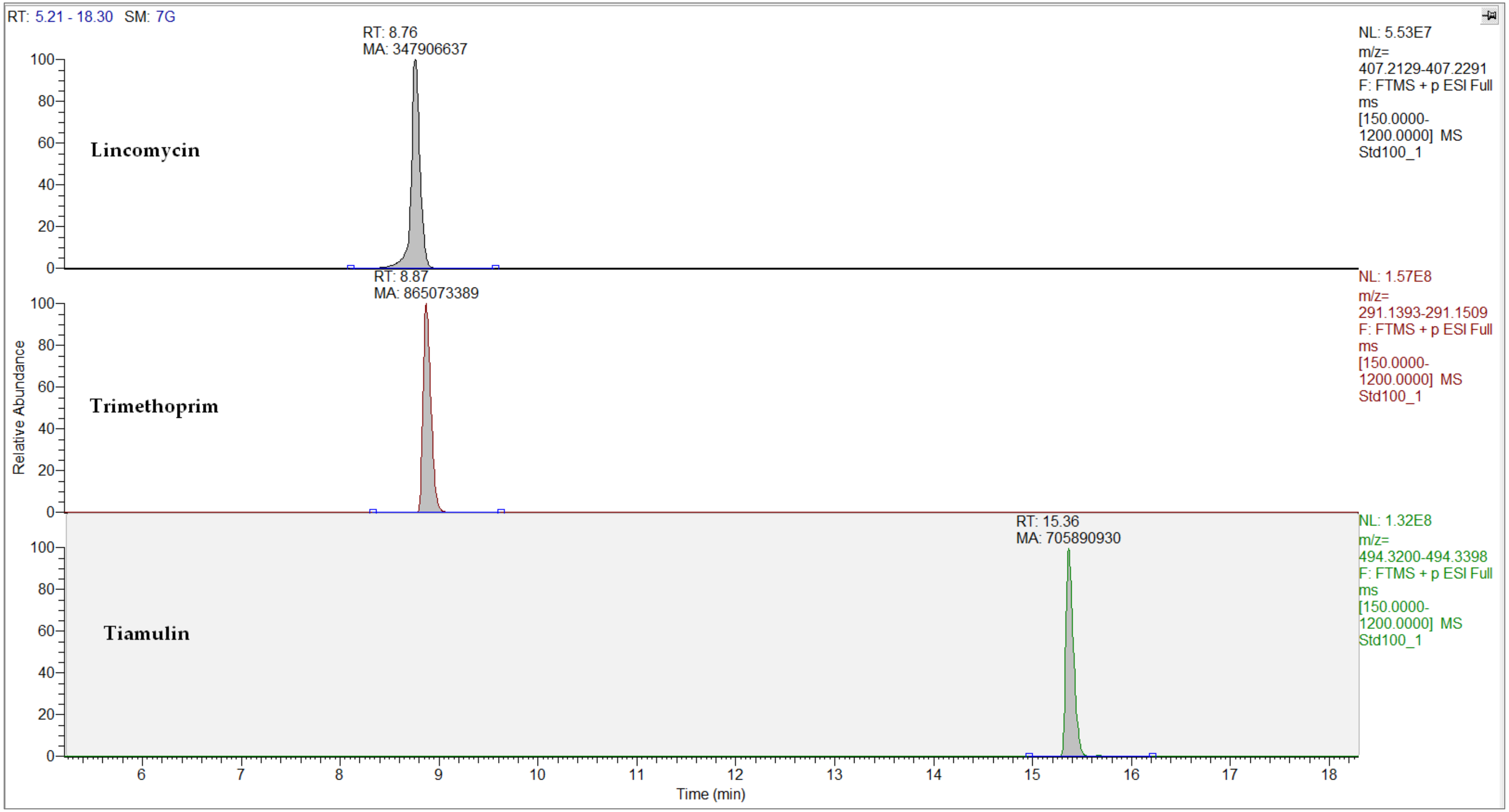

Figure S4. Extracted ion chromatograms: standard mixture of lincomycin, trimethoprim and tiamulin (100 ng mL^{-1}).

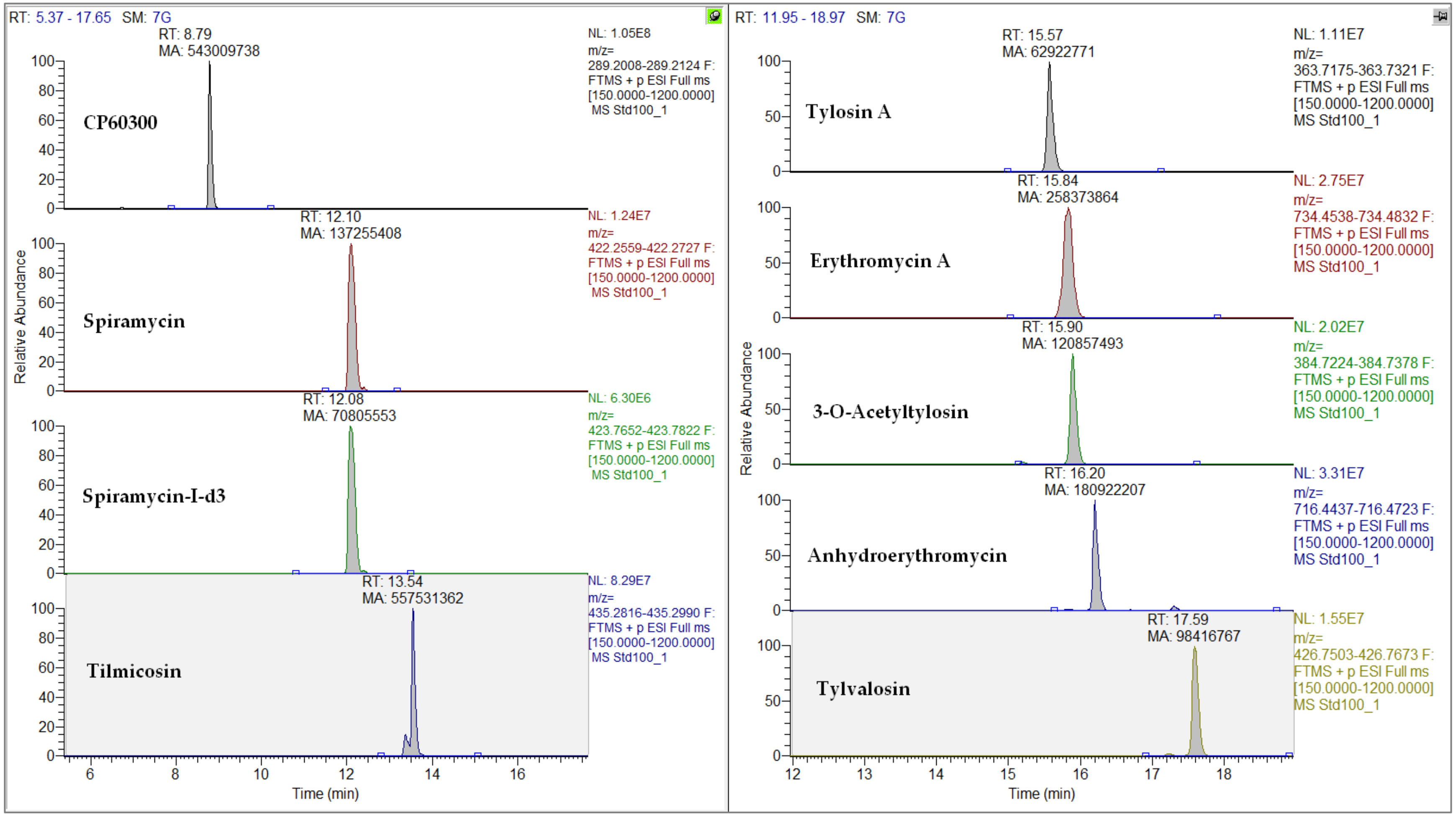

Figure S5. Extracted ion chromatograms: standard mixture of macrolides (100 ng mL^{-1}).

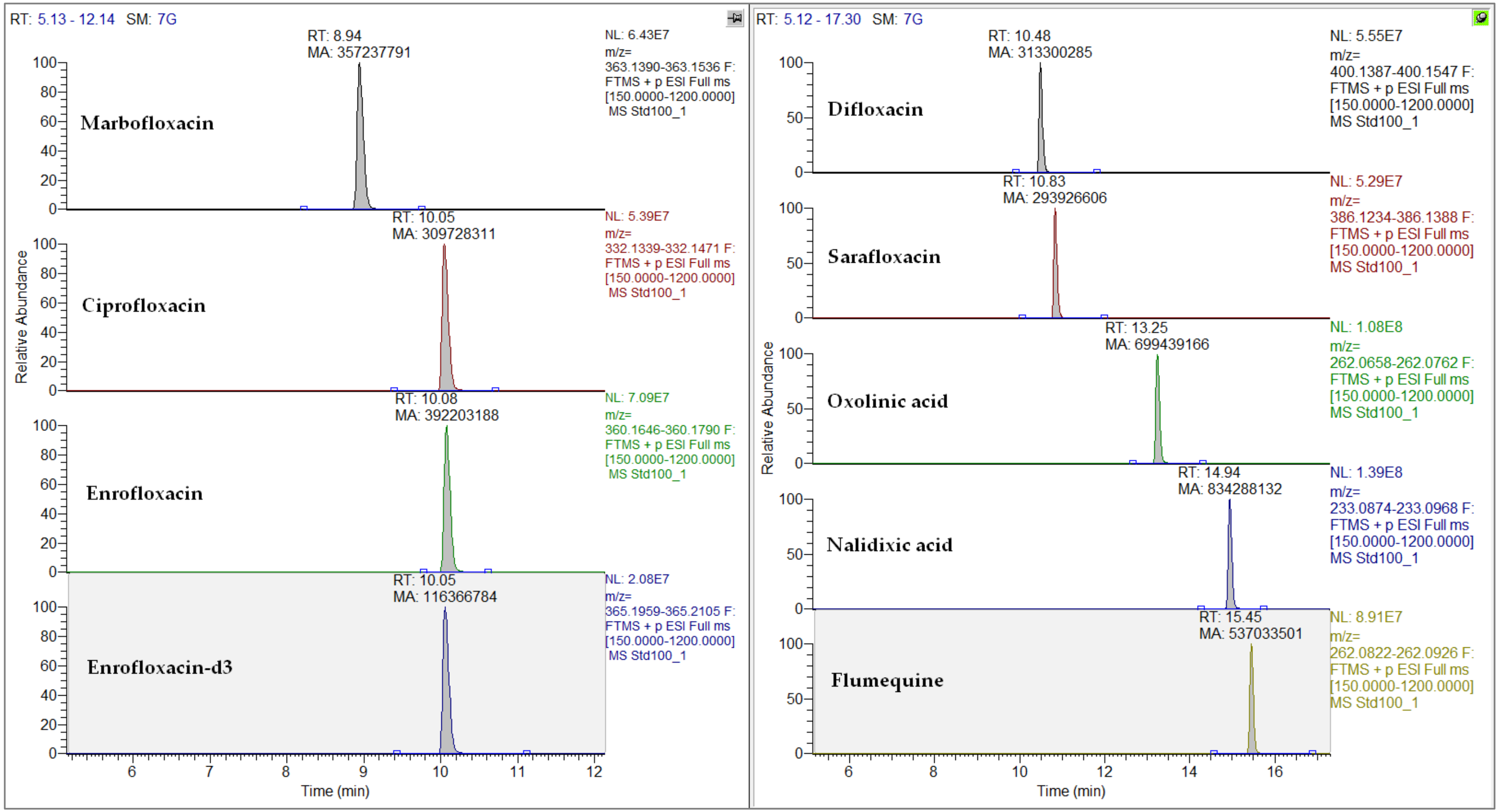

Figure S6. Extracted ion chromatograms: standard mixture of quinolones (100 ng mL^{-1}).

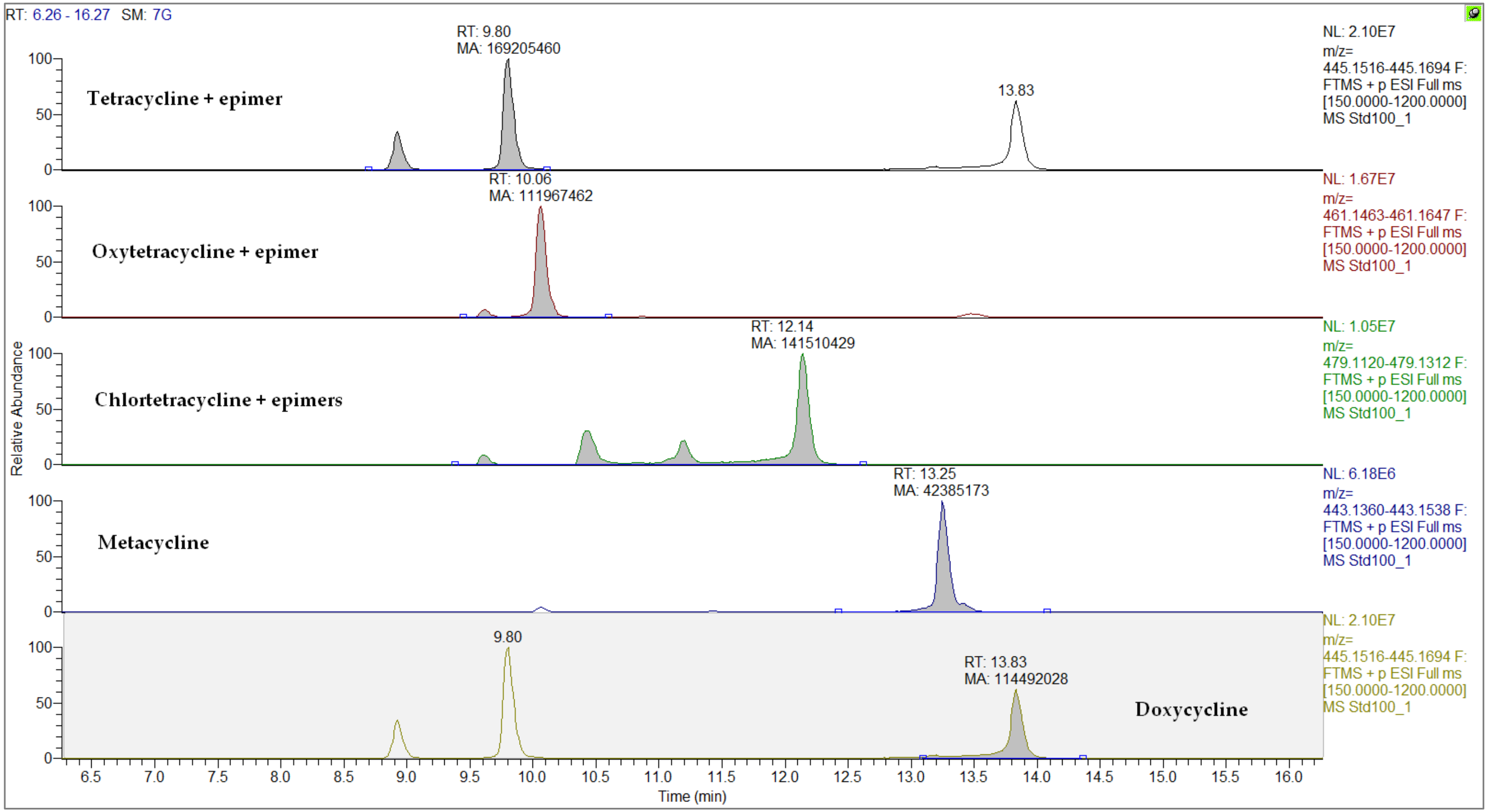

Figure S7. Extracted ion chromatograms: standard mixture of tetracyclines (100 ng mL^{-1}).

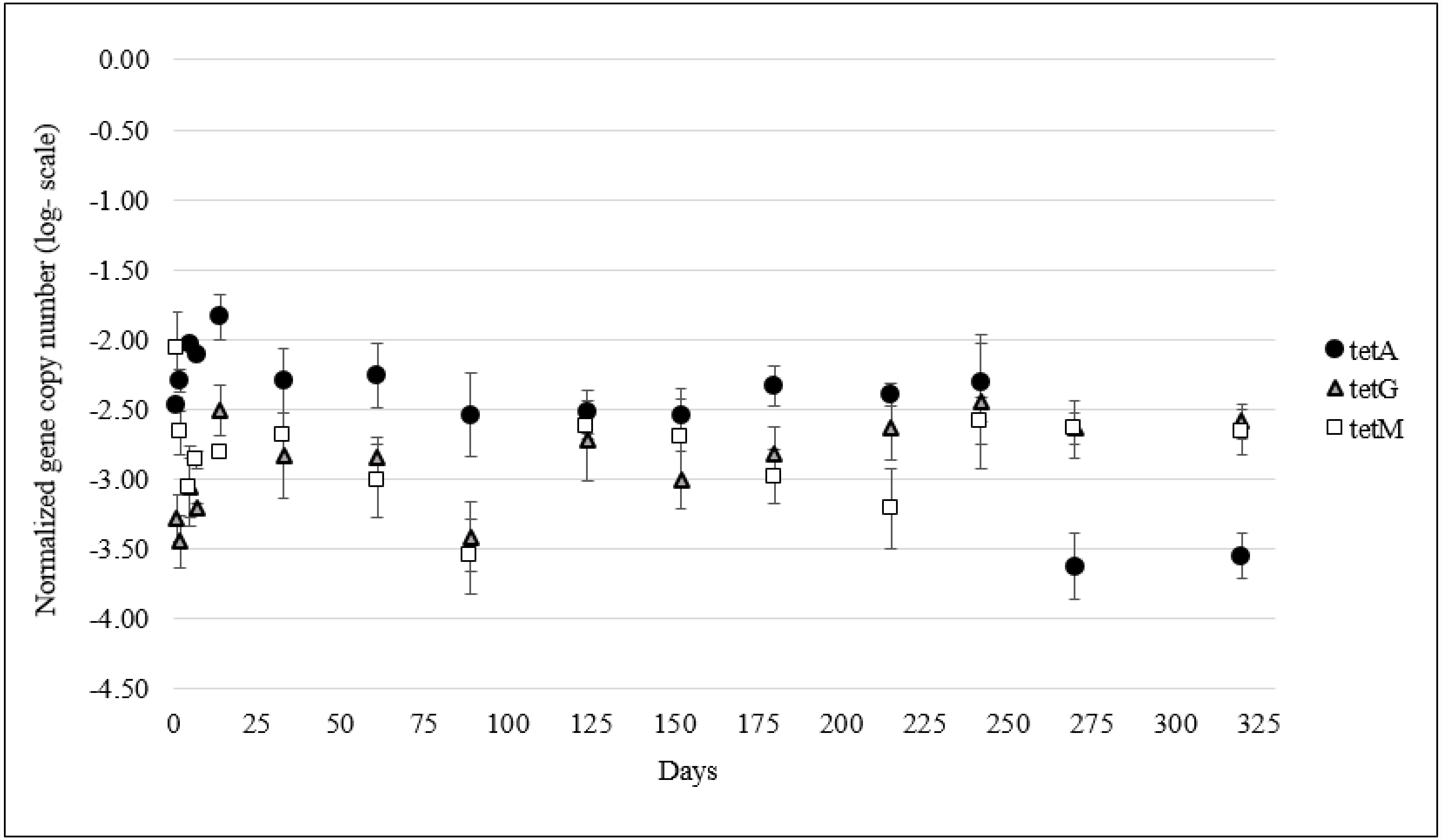

Figure S8. Relative abundances of *tetA*, *tetG* and *tetM* (copy gene number/copy number of 16S rRNA-log scale) in soil during the incubation experiment.

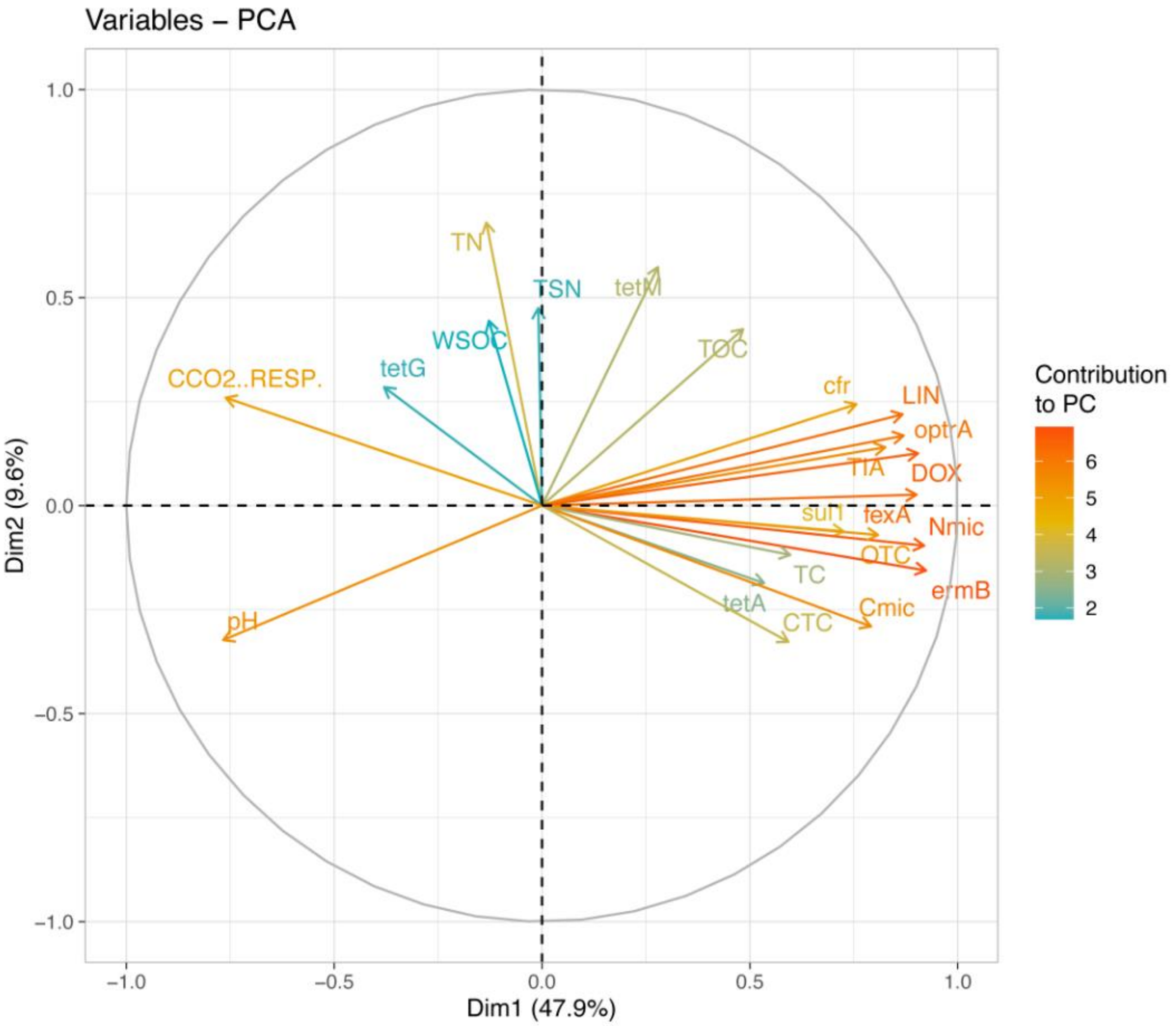

Figure S9. Loading plot of PCA.

References

1. Berendsen, B.J.; Wegh, R.S.; Memelink, J.; Zuidema, T.; Stolker, L.A. The analysis of animal faeces as a tool to monitor antibiotic usage. *Talanta* **2015**, *132*, 258–268, https://doi.org/10.1016/j.talanta.2014.09.022.
2. Berendsen, B.; Lahr, J.; Nibbeling, C.; Jansen, L.; Bongers, I.; Wipfler, E.; van de Schans, M. The persistence of a broad range of antibiotics during calve, pig and broiler manure storage. *Chemosphere* **2018**, *204*, 267–276, https://doi.org/10.1016/j.chemosphere.2018.04.042.
3. Zhi, S.; Zhou, J.; Liu, H.; Wu, H.; Zhang, Z.; Ding, Y.; Zhang, K. Simultaneous extraction and determination of 45 veterinary antibiotics in swine manure by liquid chromatography-tandem mass spectrometry. *J. Chromatogr. B* **2020**, *1154*, 122286, https://doi.org/10.1016/j.jchromb.2020.122286.
4. Argüeso-Mata, M.; Bolado, S.; Jiménez, J.J.; López-Serna, R. Determination of antibiotics and other veterinary drugs in the solid phase of pig manure. *Chemosphere* **2021**, *275*, 130039, https://doi.org/10.1016/j.chemosphere.2021.130039.
5. Comprehensive Antibiotic Resistance Database, Available online: https://card.mcmaster.ca (accessed on 13 March 2024)
6. World Health Organization,2018. Critically Important Antimicrobials for Human Medicine. 6th revision 2018, Available online: https://www.who.int/publications/i/item/9789241515528 (accessed on 13 March 2024)
7. European Medicines Agency, 2021.Sales of veterinary antimicrobial agents in 31 European countries in 2019 and 2020 - Trends from 2010 to 2020. Available online: https://www.ema.europa.eu (accessed on 13 March 2024)

Disclaimer/Publisher's Note: The statements, opinions and data contained in all publications are solely those of the individual author(s) and contributor(s) and not of MDPI and/or the editor(s). MDPI and/or the editor(s) disclaim responsibility for any injury to people or property resulting from any ideas, methods, instructions or products referred to in the content.
